# Rational design of ICD-inducing nanoparticles for cancer immunotherapy

**DOI:** 10.1126/sciadv.adk0716

**Published:** 2024-02-07

**Authors:** Zhanzhan Zhang, Zheng Pan, Qiushi Li, Qingqing Huang, Linqi Shi, Yang Liu

**Affiliations:** ^1^ College of Chemistry, Key Laboratory of Functional Polymer Materials (Ministry of Education), Nankai University, Tianjin 300071, China.; ^2^ State Key Laboratory of Medicinal Chemical Biology, Nankai University, Tianjin 300071, China.; ^3^ School of Medical Imaging, Tianjin Medical University, Tianjin 300203, China.; ^4^ Frontiers Science Center for New Organic Matter Nankai University, Tianjin 300071, China.

## Abstract

Nanoparticle-based cancer immunotherapy has shown promising therapeutic potential in clinical settings. However, current research mainly uses nanoparticles as delivery vehicles but overlooks their potential to directly modulate immune responses. Inspired by the endogenous endoplasmic reticulum (ER) stress caused by unfolded/misfolded proteins, we present a rationally designed immunogenic cell death (ICD) inducer named NanoICD, which is a nanoparticle engineered for ER targeting and retention. By carefully controlling surface composition and properties, we have obtained NanoICD that can effectively accumulate in the ER, induce ER stress, and activate ICD-associated immune responses. In addition, NanoICD is generally applicable to various proteins and enzymes to further enhance the immunomodulatory capacity, exemplified by encapsulating catalase (CAT) to obtain NanoICD/CAT, effectively alleviated immunosuppressive tumor microenvironment and induced robust antitumor immune responses in 4T1-bearing mice. This work demonstrates engineered nanostructures’ potential to autonomously regulate biological processes and provides insights into the development of advanced nanomedicines for cancer treatment.

## Teaser

A nanoparticle-based ICD inducer is designed to stimulate antitumor immune responses for cancer treatments.

## INTRODUCTION

Cancer immunotherapy has changed the paradigm of cancer treatment by mobilizing the host immune system to recognize and destroy cancer cells (*1*, *2*). Recently, nanoparticles have been increasingly used in cancer immunotherapy due to their abilities to optimize biodistribution, improve circulation stability, and reduce the side effects of therapeutic agents (*1*, *3*). To date, most current research on nano-immunotherapies uses nanoparticles as delivery vehicles. The design of nanoparticles usually focuses on prolonging circulation time and improving delivery efficiency but largely ignores the potential of nanoparticles to directly regulate the immune system (*4**–**6*). Recently, some serendipitous discoveries have suggested that certain solid nanoparticles or lipid nanoparticles with specific chemical structures, composition, or formulations are directly involved in immune regulation (*7**–**12*). However, rationally designed nanoparticles with immunomodulatory capacity have not been reported so far. Nanoparticles are a special class of materials with unique particle sizes and highly engineerable surfaces (*13*). These properties allow tailor-made nanoparticles to target specific tissues, cells, and even organelles (*14**–**16*). Moreover, the binding affinity between the nanoparticle and its target can be finely controlled by tuning nano-multivalent interactions, thus offering nanoparticles great potential to modulate immune systems (*17*, *18*).

Currently, most cancer immunotherapies are designed on the basis of the cancer immune cycle, which is a self-propagating process that elicits effective antitumor immune responses (*19*). However, low immune cell infiltration and immunosuppressive networks in tumor microenvironment (TME) render tumors less immunogenic and severely suppress host immune responses (*20*). Recent studies have shown that neoplastic cells undergoing immunogenic cell death (ICD) exert a vaccine-like function to generate antitumor immunity, thereby transforming a “cold” tumor into an immunogenic “hot” tumor (*21**–**23*). ICD is considered a stress-induced process in which endoplasmic reticulum (ER) stress is required for the release of tumor-associated antigens (TAAs) and damage-associated molecular patterns (DAMPs) from tumor cells to provide antigenicity and adjuvanticity, respectively (*24**–**26*). Recently, several clinical trials have shown that pretreatment with ICD inducers notably improves the response rate and survival of immunotherapies based on checkpoint blockade (*27*). To date, many ICD induction strategies have been developed, including treatment with chemical drugs, such as anthracyclines, chemical protein phosphatase 1/GADD34 inhibitors, etc., and physical induction strategies, such as photodynamic therapy and radiotherapy (*24*, *28*, *29*). Recently, nanoparticle-based ICD inducers with high ICD-inducing efficacy and low side effects have emerged (*26*). However, most nanoparticle-based ICD inducers are chemical inducers or photosensitizers delivered by nano-sized drug carriers. In these strategies, the ICD is induced by the payload, while the nanomaterial has no immunomodulatory function.

**Fig. 1.**
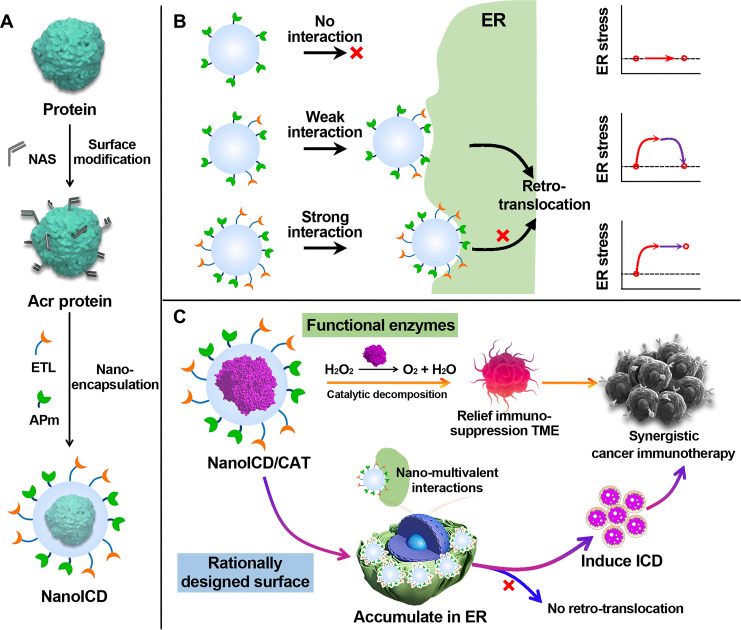
Schematic representation of the synthesis and the mechanism of NanoICD to induce ICD. (**A**) Schematic illustration of the synthesis of NanoICD; (**B**) the mechanism by which NanoICD induces ER stress; (**C**) encapsulated CAT and functional polymer surface of NanoICD/CAT synergistically regulate immune system for cancer treatment. NanoICD with strong surface interaction with ER hinders the retro-translocation process and effectively induces ER stress.

Given the relationship between ER stress and ICD induction, disturbing ER homeostasis is essential for successful ICD induction (*24*, *30*). In normal cells, endogenous signals such as the accumulation of unfolded or misfolded proteins in the ER lumen can disturb ER proteostasis and induce ER stress (*31*, *32*). However, the ER subtly relieves this stress by retro-translocating these proteins to the cytoplasm and activating proteasomes for degradation (*31**–**33*). Mimicking this accumulation process but avoiding the subsequent stress relief should be an effective strategy to induce ER stress. Therefore, we hypothesize that synthetic nanoparticles with the ability to target and retain in ER can effectively induce ER stress and activate ICD-associated immune responses. As a proof of concept, we present here a rationally designed ICD inducer based on nanoparticles engineered for ER targeting and ER retention (denoted as NanoICD, Fig. 1). NanoICD was synthesized by a protein nanoencapsulation method (*15*, *34*, *35*), which involved in situ–copolymerized neutral monomers [acrylamide (AAm)], positively charged monomers {*N*-(3-aminopropyl)-methacrylamine [APm]} (*36*), ER-targeting ligand {*N*-(2-((4-methylphenyl)sulfonamido)ethyl)acrylamide [ETL]} (*37*), and the cross-linkers [*N,N*′-methylenebisacrylamide (BIS)] on the surface of each protein molecule (Fig. 1A). The introduction of APm provides a positively charged surface for NanoICD, which ensures the effective uptake of NanoICD by cancer cells and the subsequent escape from lysosomes. Meanwhile, the integration of multiple ETLs allows NanoICD to target and tightly bind to the ER after the lysosomal escape. By controlling monomer compositions and formulation methodologies, NanoICD efficiently accumulates in the ER, induces ER stress, and eventually activates ICD-associated immune responses (Fig. 1B). In this work, immunologically inert bovine serum albumin (BSA) was first used for the nanoencapsulation (NanoICD/BSA) to better understand the immunomodulatory function of the polymer surface. Furthermore, the BSA can be replaced with other proteins and enzymes to provide additional functions to cooperate with the ICD-inducing function of the polymer surface. As a demonstration, catalase (CAT) was used to perform the nanoencapsulation to synthesize NanoICD/CAT (Fig. 1C). In addition to the induction of ICD of tumor cells, NanoICD/CAT effectively alleviated hypoxic and inflammatory TME by decomposing hydrogen peroxide (H_2_O_2_) to oxygen (O_2_), resulting in the relief of tumor immunosuppression and further enhancing the antitumor immune responses (*38**–**40*). Together, these unique features make NanoICD an innovative strategy for effective cancer immunotherapy. This work demonstrates that a nanoparticle with a rationally designed surface can directly modulate the immune system without the aid of conventional immunomodulating drugs, providing new insights into the design of advanced nanomedicines for cancer immunotherapy.

## RESULTS

### The relationship between the amount of ETL on NanoICD and its ability to induce ICD

**Fig. 2.**
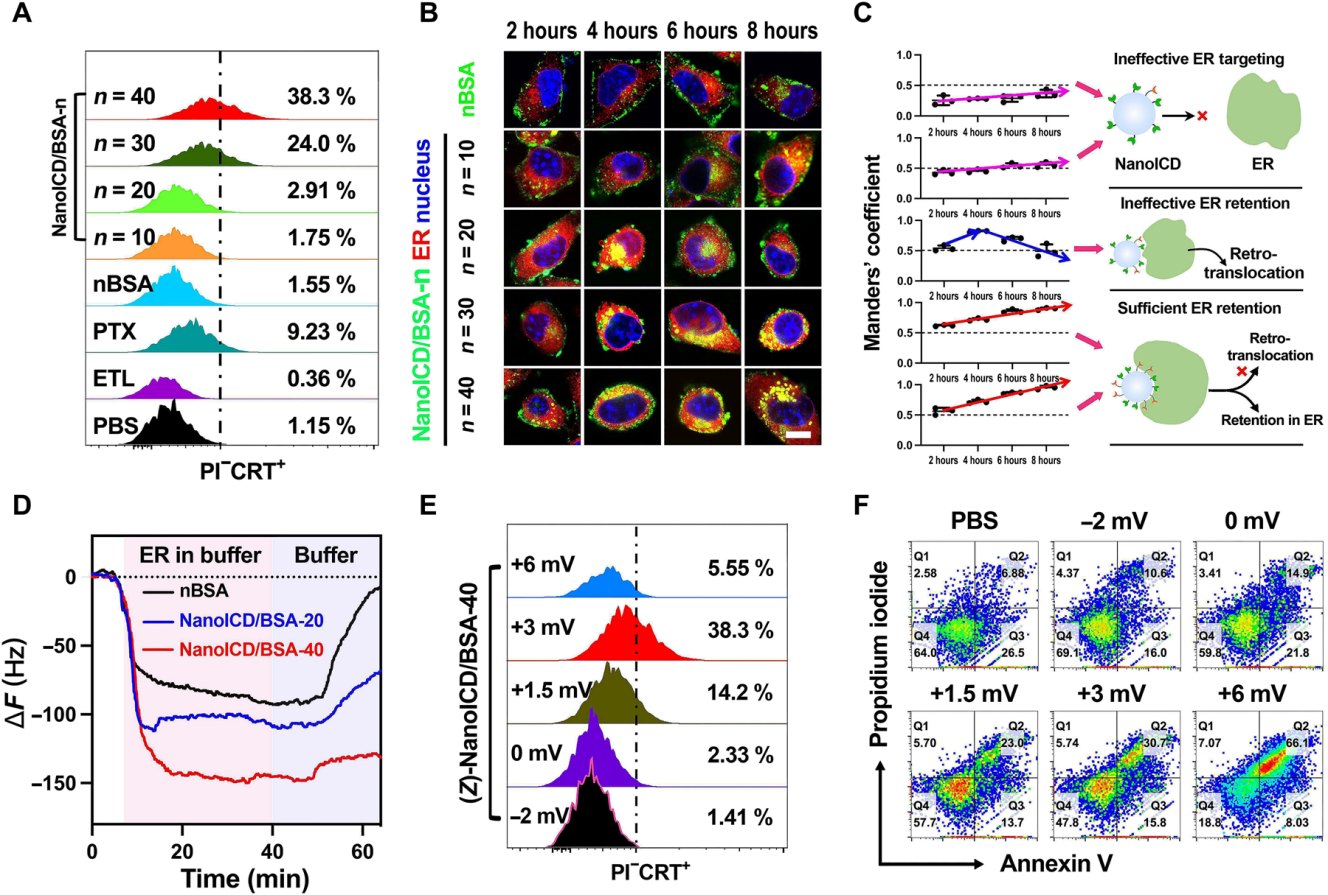
The relationship between the amount of ETL/APm and the ICD-inducing activity of NanoICD/BSA. (**A**) Pre-apoptotic CRT exposure on cell (B16F10) surface after the treatment with PBS, ETL, PTX, nBSA, and NanoICD/BSA-*n* (*n* = 10, 20, 30, and 40). (**B**) CLSM images showing the ER targeting and retention capabilities of nBSA and NanoICD/BSA-*n* (*n* = 10, 20, 30, 40) in B16F10 cells. (**C**) Quantitative analysis of the colocalization of nBSA and NanoICD/BSA-*n* with ER. Manders’ coefficient M2 indicates the fraction of NanoICD overlapping ER. (**D**) QCM-D experiment showing the binding affinities between nBSA, NanoICD/BSA-*n* (*n* = 20, 40), and ER. (**E**) Pre-apoptotic CRT exposure on the cell (B16F10) surface after treatment with (*Z*)-NanoICD/BSA-40 (*Z* = −2, 0, 1.5, 3, and 6 mV). (**F**) Apoptosis of B16F10 cells after treatment with (*Z*)-NanoICD/BSA-40 (*Z* = −2, 0, 1.5, 3, and 6 mV). Scale bar, 10 μm.

To achieve ER accumulation, nanoparticles must first enter the cytoplasm and then bind to the ER. For NanoICD, cell internalization and ER targeting ability are achieved by introducing APm and ETL during the polymerization to provide positive surface charge and ER-targeting groups, respectively. In particular, the amount of ETL determines the mass of NanoICD bound to ER and ultimately affects the efficiency of ICD induction. To investigate the relationship between the amount of ETL on NanoICD and its efficiency in inducing ICD, we synthesized a series of NanoICD/BSA (with similar zeta potential, ~3 mV; details in table S2) containing different amounts of ETL (denoted as NanoICD/BSA*-n*, where *n* represents the amount of ETL on each NanoICD). Since the pre-apoptotic calreticulin (CRT) exposure on the cell surface is the most relevant DAMP for ICD (*5*, *30*), the level of surface CRT exposure was used as the standard criterion to evaluate the ability of these NanoICD/BSA to induce ICD. In this study, all these NanoICD/BSA were incubated with mouse melanoma cells (B16F10) for 12 hours. Phosphate-buffered saline (PBS)– and paclitaxel (PTX)–treated cells were used as negative and positive controls, respectively. For better demonstration, a free form of ETL and a nanoparticle similar to NanoICD/BSA but without ETL (denoted as nBSA) were used as comparative groups. After incubation, cells were collected and stained with ATTO-488-CRT and propidium iodide (PI) for flow cytometric analysis. As shown in Fig. 2A, negligible pre-apoptotic CRT exposure (gated on PI^−^CRT^+^) was observed from cells treated with nBSA (1.55%), NanoICD/BSA-10 (1.75%), and NanoICD/BSA-20 (2.91%) compared to the negative control (PBS-treated cells, 1.15%). In contrast, the cells treated with NanoICD/BSA-30 (24%) and NanoICD/BSA-40 (38.3%) exhibit a significant amount of PI^−^CRT^+^ cells. This result indicates that high surface density of ETL is required for NanoICD/BSA to effectively induce ER stress. Moreover, the free form of ETL did not induce any CRT exposure (0.36%), even at a much higher dosage. These results suggest that strong multivalent binding to ER is essential for NanoICD/BSA to effectively trigger ER stress and induce ICD.

Next, we investigated the mechanism underlying the ICD-inducing activity of NanoICD/BSA. As shown in figs. S6 and S7, all these NanoICD/BSA-*n* were efficiently internalized by B16F10 cells and escaped from endosomes within 2 hours. This result suggests that the different ICD-inducing performance of NanoICD/BSA-*n* was not affected by their cell internalization ability but may be caused by their different ER-targeting and retention ability. For the verification, the intracellular distribution of NanoICD/BSA-*n* was observed by a confocal laser scanning microscope (CLSM) at different time points. As shown in Fig. 2B, the nBSA and NanoICD/BSA-10 were mainly distributed in the cytoplasm, and very few of them were observed in the ER after 8 hours of incubation. NanoICD/BSA-20–treated cells exhibited obvious colocalization of NanoICD/BSA-20 with ER at 4 hours, but the colocalization significantly decreased, and the NanoICD/BSA-20 were transported back to the cytoplasm at 6 and 8 hours. Despite the successful targeting to the ER, NanoICD/BSA-20 failed to survive from the retro-translocation effect of ER. Considering the ineffectiveness of NanoICD/BSA-20 in inducing pre-apoptotic CRT exposure of cells, this result suggests that long-term retention and accumulation of the nanoparticles in ER may be essential for the induction of ICD. This is confirmed by the results from the cells treated with NanoICD/BSA-30 and NanoICD/BSA-40, where the cells showed significant pre-apoptotic CRT exposure (Fig. 2A), and the nanoparticles were mainly distributed in the ER, and their ER accumulation increased along with the incubation time. These results were further confirmed by quantitative analysis of the colocalization of the NanoICD/BSA-*n* with ER by using Manders’ coefficients M2 (fraction of NanoICD/BSA overlapping ER) (*41*). As shown in Fig. 2C, M2 of cells treated with nBSA and NanoICD/BSA-10 were lower than 0.6 at all four time points, suggesting that low surface density of ETL (<10 per nanoparticle) is insufficient for NanoICD to achieve effective ER targeting. NanoICD/BSA-20–treated cells exhibited a much higher M2 (0.82) at 4 hours but gradually decreased to 0.49 after 8 hours of incubation, suggesting that moderate density of ETL (~20 per nanoparticle) enhanced the interaction of NanoICD with ER, but the interaction was not strong enough to overcome the retro-translocation to achieve long-term retention and accumulation in ER. Of note, the M2 of cells treated with NanoICD/BSA-30 (0.86 at 6 hours, 0.90 at 8 hours) and NanoICD/BSA-40 (0.86 at 6 hours, 0.95 at 8 hours) was significantly higher than other treatments and increased along with the incubation time, suggesting that a high density of ETL (>30 per nanoparticle) is required for NanoICD/BSA to effectively interact with ER, overcome the retro-translocation to be retained in ER, and eventually induce ICD of the cell.

To verify this hypothesis, we then determined the mass of NanoICD/BSA bound to ER using a quartz crystal microbalance with dissipation monitoring (QCM-D) (*42*). Three NanoICD/BSA-*n* with different amounts of ETL, including 0 (nBSA), 20 (NanoICD/BSA-20), and 40 (NanoICD/BSA-40), were used in this assay. Briefly, nBSA and NanoICD/BSA (100 μg/ml) were first conjugated to the Au sensor chip via Traut’s reagent and washed with PBS buffers before exposure to freshly extracted ER (details in the Supplementary Materials). After the binding reached equilibrium, the flow phase was replaced with PBS to simulate the retro-translocation process of the ER. The frequency shifts of QCM-D were continuously monitored, and the results are summarized in Fig. 2D. According to the results, NanoICD/BSA-40 (Δ*F* = 149.85 Hz) exhibited a significantly higher maximum frequency shift than that of NanoICD/BSA-20 (Δ*F* = 110.14 Hz) and nBSA (Δ*F* = 91.68 Hz), indicating that the high density of ETL confers a strong binding between NanoICD/BSA-40 and the ER. When rinsed with PBS, only a slight change in frequency shift was observed in NanoICD/BSA-40 groups (Δ*F* = 130.8 Hz, ΔΔ*F* = 19.05 Hz) compared to NanoICD/BSA-20 (Δ*F* = 68.2 Hz, ΔΔ*F* = 41.94 Hz) and nBSA groups (Δ*F* = 7 Hz, ΔΔ*F* = 84.67 Hz), indicating that the strong binding of NanoICD/BSA-40 offers the potential to overcome the retro-translocation to retain in the ER.

### The relationship between the amount of APm on NanoICD and its ability to induce ICD

Zeta potential is another factor that affects the ICD-inducing ability of NanoICD. By increasing the amount of APm during the polymerization or neutralizing APm with succinic anhydride (SA) (*20*), we obtained a series of NanoICD/BSA with similar ETL amount (~40) but different zeta potentials [ranging from −2 to +6 mV, denoted as (*Z*)-NanoICD/BSA, where *Z* represents the zeta potential of each NanoICD/BSA; details in table S1]. The ICD-inducing capabilities of these (*Z*)-NanoICD were evaluated by flow cytometric analysis. As shown in Fig. 2E, a significantly reduced population of PI^−^CRT^+^ cells were detected in the cells treated with (+1.5)-NanoICD/BSA (14.2%) compared to (+3)-NanoICD/BSA (38.3%). In addition, the amount of PI^−^CRT^+^ cells were almost undetectable when the cells were treated with (0)-NanoICD/BSA (2.33%) and (−2)-NanoICD/BSA (1.41%). The significantly reduced ICD-inducing capacity of NanoICD/BSA may be attributed to its reduced cellular uptake efficiency as the decrease in zeta potential (fig. S6), indicating that effective cellular uptake is a prerequisite for NanoICD/BSA to induce ICD. However, although (+6)-NanoICD/BSA showed the highest cellular uptake, only a small amount of PI^−^CRT^+^ cells were detected from the cells treated with (+6)-NanoICD/BSA (5.55%). In addition, (+6)-NanoICD/BSA exhibited much higher cytotoxicity than (+3)-NanoICD/BSA (fig. S8). These results imply that (+6)-NanoICD/BSA might behave differently from (+3)-NanoICD/BSA after cellular uptake. To investigate, we next performed an annexin V (AV)–PI–based apoptosis assay on the NanoICD/BSA-treated cells. As shown in Fig. 2F, the amount of late apoptotic cells (AV^+^PI^+^) was significantly increased from 30.7% of (+3)-NanoICD/BSA to 66.1% of (+6)-NanoICD/BSA, indicating that (+6)-NanoICD/BSA induced cells to undergo apoptosis rather than ICD. Therefore, an appropriate zeta potential (~3 mV) to allow efficient cellular uptake and endosomal escape while avoiding excessive cytotoxicity is critical for NanoICD/BSA to effectively activate ICD-associated immune response. In the following, NanoICD/BSA will be explicitly referred to as NanoICD/BSA with surface ETL groups of ~40 and a zeta potential of ~3 mV, unless otherwise noted.

**Fig. 3.**
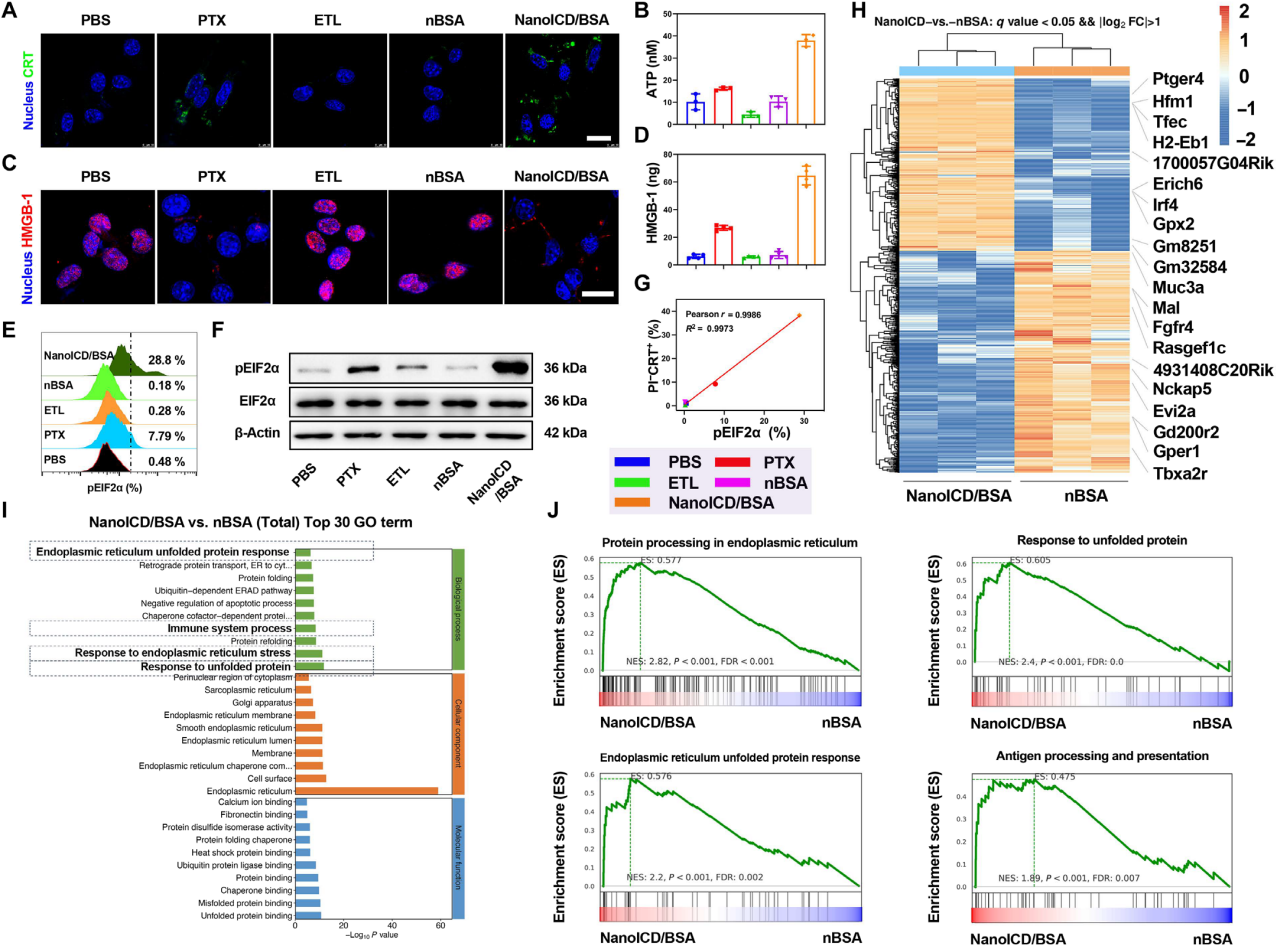
The potential mechanism of NanoICD/BSA to induce ICD of tumor cells. (**A**) CLSM images show the exposure of CRT on B16F10 surface after treatment with PBS, PTX, ETL, nBSA, and NanoICD/BSA. (**B**) ATP levels in cellular supernatants after different treatments. (**C**) The exodus of HMGB-1 from cell nuclei after different treatments. (**D**) Extracellular concentrations of HMGB-1 after treating the cells with PBS, PTX, ETL, nBSA, and NanoICD/BSA. (**E**) pEIF2α levels in B16F10 cells after treatment with PBS, PTX, ETL, nBSA, and NanoICD/BSA. (**F**) Western blot analysis of the expression of β-actin, EIF2α, and pEIF2α in B16F10 cells after different treatments. (**G**) The correlations between pEIF2α levels and pro-apoptotic CRT on cell surface after the treatment. (**H**) Heatmap and hierarchical clustering analysis of DEGs of B16F10 after different treatments. (**I**) Top 30 enriched pathways in GO analysis in NanoICD/BSA-treated B16F10 cells. (**J**) GSEA shows the up-regulation of protein processing in ER, response to unfolded protein, the ER UPR, and antigen processing and presentation pathways in NanoICD/BSA-treated B16F10 cells. Data are presented as means ± SD from three independent experiments (biological replicates, *n* = 3). Scale bars, 20 μm.

To further investigate the ICD-inducing activity of NanoICD/BSA, CLSM-based analysis was performed to directly observe the pre-apoptotic exposure of CRT on the cell surface after treatment. PBS, PTX, ETL, and nBSA were used as comparison groups for better demonstration. As shown in Fig. 3A, both PTX- and NanoICD/BSA-treated cells showed significant surface CRT exposure. In contrast, negligible CRT exposure was observed in cells treated with PBS, ETL, and nBSA, which is consistent with the results of the flow cytometric analysis (Fig. 2A). In addition to surface CRT exposure, two other important indicators of ICD, including the secretion of adenosine triphosphate (ATP) and the post-apoptotic exodus of high mobility group box 1 (HMGB-1), were also evaluated (*24*). As shown in Fig. 3B, significantly higher levels of ATP were detected in cellular supernatants after treatment with NanoICD/BSA and PTX than those of PBS, ETL, and nBSA. CLSM- and enzyme-linked immunosorbent assay (ELISA)–based analyses were then performed to investigate the intracellular and extracellular levels of HMGB-1 after different treatments. As shown in Fig. 3 (C and D), the decrease in intracellular HMGB-1 levels and increase in extracellular HMGB-1 levels were observed from the cells treated with PTX and NanoICD/BSA, indicating the exodus of HMGB-1 after treatment. Together, the observation of these three important indicators of ICD suggests the great potential of NanoICD/BSA as an effective ICD inducer.

### The potential mechanism of NanoICD/BSA to induce ICD

Next, we investigated the mechanism behind this ICD-inducing ability of NanoICD/BSA. Since NanoICD/BSA that can effectively trigger ICD-associated immune responses showed efficient ER targeting and retention, we hypothesized that the long-term retention of NanoICD/BSA in the ER might induce ER stress and the phosphorylation of eukaryotic translation initiation factor 2α (EIF2α). For verification, the phosphorylation of EIF2α (pEIF2α) in cells was evaluated by flow cytometry–based analysis after different treatments. As shown in Fig. 3E, negligible changes in pEIF2α levels were observed from the cells treated with ETL (0.28%) and nBSA (0.18%). In contrast, pEIF2α levels were significantly up-regulated in PTX (7.79%)– and NanoICD/BSA-treated cells (28.8%), suggesting that NanoICD effectively induced ER stress. Similar results were observed from the Western blot–based analysis (Fig. 3F), where NanoICD/BSA-treated cells showed the highest levels of pEIF2α. Last, these samples were subjected to correlation analysis between pEIF2α levels and surface CRT exposure, which revealed a strong positive linear correlation (Pearson’s *r* = 0.9986, *R*^2^ = 0.9973) (Fig. 3G). These results suggest that NanoICD/BSA induces ICD-associated immunogenicity by promoting ER stress and pEIF2α.

In addition, we also performed whole RNA sequencing to compare gene expression levels in B16F10 cells after NanoICD/BSA, nBSA, and PBS treatment for 24 hours. As shown in figs. S9 to S11, principal components analysis revealed that differential gene expressions were closely correlated with the different treatments, indicating that nanoparticles with different surface properties could modulate the gene expression of B16F10. Further analysis of differentially expressed genes (DEGs) revealed that 450 genes were significantly up-regulated, and 484 genes were significantly down-regulated in NanoICD/BSA-treated cells compared to those treated with nBSA (Fig. 3H and fig. S12). Gene Ontology (GO), Kyoto Encyclopedia of Genes and Genomes (KEGG), and Gene Set Enrichment Analyses (GSEA) were then performed to investigate the biological functions of these DEGs. As expected, the ER unfolded protein response (ER UPR), immune system process, response to ER stress, and response to unfolded protein pathways were among the top up-regulated GO terms in NanoICD-treated cells (Fig. 3, I and J), and protein processing in ER and antigen processing and presentation pathways were among the top up-regulated KEGG terms (fig. S13 and Fig. 3J). These up-regulated gene terms suggest that NanoICD/BSA acts as a biomimetic unfolded/misfolded protein that induces ER stress and ER UPR, promotes antigen processing and presentation, and ultimately activates immune system processes.

### ICD-associated immune responses induced by NanoICD/BSA

During the development of tumors, tumor cells transmit a “don’t eat me” signal through the up-regulation of CD47 and its binding to signal regulatory protein α (SIRPα) on the surface of antigen-presenting cells (APCs) via the CD47-SIRPα pathway. This interaction effectively hinders the immune clearance of cancer cells. For a typical ICD, the surface-exposed CRT acts as an “eat me” signal, mediated by the CRT–low density lipoprotein receptor (LPR1, CRT-LPR1) pathway, to promote the recognition and phagocytosis of TAAs by APCs (*43*). For demonstration, B16F10 (prestained with DiD) were first incubated with PBS, PTX, ETL, nBSA, or NanoICD/BSA and then cocultured with bone marrow–derived dendritic cells (BMDCs, prestained with DiO) for flow cytometric analysis. As shown in fig. S14, NanoICD/BSA treatment (45.1%) significantly enhanced the phagocytosis of B16F10 by BMDCs compared to those treated with PBS (5.45%), ETL (8.33%), nBSA (9.43%), and even PTX (13.9%), indicating that NanoICD/BSA effectively promoted BMDC-mediated phagocytosis of tumor cells. After the phagocytosis, the cross-presentation of TAAs was further studied. This was achieved by using ovalbumin (OVA)–expressing B16F10 cells (B16F10-OVA) to perform a similar study as above. As shown in fig. S15, NanoICD/BSA-treated BMDCs exhibited the highest levels of SIINFEKL-H2kb complex (22.9%) compared to other treatments, suggesting that NanoICD/BSA effectively promoted the cross-presentation of OVA peptide on the major histocompatibility protein I (MHC I) complex. In addition, the exodus of HMGB-1 and the secretion of ATP during ICD also serve as adjuvants to promote APC maturation (*26*). To this end, the proportion of mature BMDCs (CD11c^+^CD80^+^CD86^+^) was investigated by coculturing BMDCs with the B16F10 cells treated with NanoICD/BSA, nBSA, ETL, PTX, and PBS. As shown in fig. S16, significantly enhanced BMDC maturation was observed from cells treated with NanoICD/BSA (43.5%) compared to those treated with PBS (16.2%), PTX (23.3%), ETL (19.1%), and nBSA (20.9%), indicating the high efficiency of NanoICD/BSA in promoting DC maturation. Quantitative analysis further confirmed these results, where the highest levels of phagocytosis, antigen presentation, and maturation of BMDCs were observed from cells treated with NanoICD/BSA. All these results suggest the great potential of NanoICD/BSA as an effective ICD inducer.

**Fig. 4.**
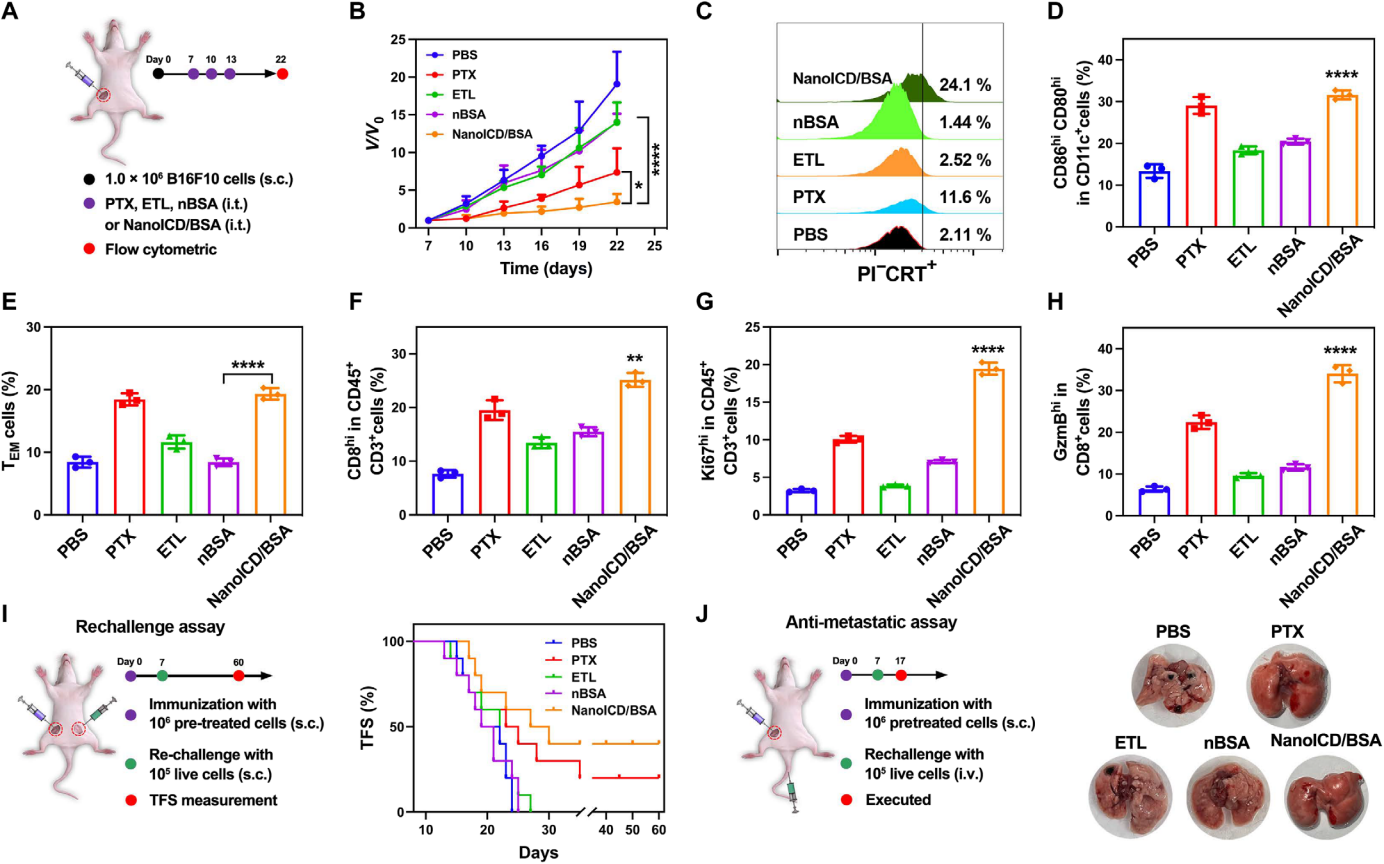
NanoICD activates ICD-associated antitumor immunity in vivo. (**A**) Schematic illustration of the experimental design. (**B**) Average tumor growth kinetics in different groups (PBS, PTX, ETL, nBSA, and NanoICD/BSA). (**C**) Pre-apoptotic CRT exposure on tumors from mice treated with PBS, ETL, PTX, nBSA, and NanoICD/BSA. (**D** and **E**) The population of CD80^+^CD86^+^ DC cells (gated on CD11c^+^) in tumor-draining lymph nodes (D) and CD44^+^CD62L^−^ effector memory cells (gated on CD3^+^CD8^+^) in spleen (E) from mice treated with PBS, PTX, ETL, nBSA, and NanoICD/BSA. (**F** to **H**) The population of TILs (F), Ki67^+^CD3^+^ (gating on CD45^+^) (G), and GzmB^+^CD8^+^ (gating on CD45^+^CD3^+^) (H) in tumors from mice treated with PBS, PTX, ETL, nBSA, and NanoICD/BSA. (**I**) Schematic illustration of the establishment of the vaccination model to evaluate the ability of NanoICD/BSA to against tumor recurrence (left), and the tumor free survival (TFS) of C57BL/6 mice after inoculation of pre-treated B16F10 cells (PBS, PTX, ETL, nBSA, or NanoICD/BSA for 24 hours), followed by the inoculation of live B16F10 cells at the contralateral flank, n = 10 (right). (**J**) Schematic illustration of the establishment of the vaccination model to evaluate the ability of NanoICD/BSA to against tumor lung metastases (left), and the lung metastases in mice after the indicated treatments (right). Data are presented as means ± SD from six independent experiments for (B) (biological replicates, *n* = 6) and three independent experiments for (D) to (H) (biological replicates, *n* = 3). Significant levels are *P < 0.05, **P < 0.01, and ****P < 0.0001.

To evaluate the potential of NanoICD/BSA to induce ICD-associated immune responses and promote antitumor immunity in vivo, the antitumor efficacy of NanoICD/BSA was studied in B16F10-bearing C57BL/6 mice. Seven days after inoculation with B16F10 cells, tumor-bearing mice were randomly divided into five groups (*n* = 6) and then intratumorally injected with PBS, PTX, ETL, nBSA, or NanoICD/BSA. The tumor volumes were continuously monitored for 22 days (as illustrated in Fig. 4A; details in the Supplementary Materials). As shown in Fig. 4B, treatment with PTX and NanoICD/BSA significantly inhibited the growth of B16F10 tumors. This significantly enhanced the idea that the antitumor effect should be attributed to the activation of ICD-associated antitumor immunity. For verification, typical ICD-related indicators, including the pre-apoptotic surface CRT exposure on tumor cells, mature DCs in tumor-draining lymph nodes, and effector memory cells in the spleen, were analyzed. As shown in Fig. 4C, treatment with NanoICD/BSA (24.1%) resulted in a significant increase in the pre-apoptotic surface CRT exposure on the cell surface compared to PBS (2.11%), ETL (2.52%), nBSA (1.44%), and PTX (11.6%) treatments, suggesting efficient activation of ICD. Similar trends were observed from the evaluation of serum HMGB-1 levels (fig. S17), where PTX- and NanoICD/BSA-treated mice exhibited significantly higher HMGB1 levels in their serum compared to those treated with PBS, ETL, and nBSA, which is consistent with the results obtained from in vitro HMGB-1 analysis (Fig. 3D). Further flow cytometric analysis of mature DCs and effector memory cells confirmed this result, with NanoICD/BSA-treated mice showing significantly higher levels of mature DCs (Fig. 4D and fig. S18; CD80^hi^CD86^hi^ cells, 32.7% for NanoICD/BSA and 28.9% for PTX) and effector memory cells (Fig. 4E and fig. S19; CD44^hi^CD62L^−^, 19.2% for NanoICD/BSA and 19.5% for PTX) than those receiving other treatments, indicating the activation of ICD-associated antitumor immune responses.

Next, we analyzed the tumor-infiltrating lymphocyte (TIL) population in tumors to further investigate the immune activation achieved by NanoICD/BSA. As shown in Fig. 4F and fig. S20, the highest level of CD8^+^ TIL (CD45^+^CD3^+^ cells) was observed in tumors from mice treated with NanoICD/BSA (26.6%) compared to PBS (8.21%), ETL (12.9%), nBSA (15.4%), and PTX (21.6%). Similar results were observed when analyzing the proliferation and activity of TILs, in which NanoICD/BSA-treated mice exhibited a significantly higher level of Ki67^hi^CD3^+^ T cells (Fig. 4G and fig. S21; CD45^+^ cells, 20.3% for NanoICD/BSA and 10.5% for PTX) and GzmB^hi^CD8^+^ T cells (Fig. 4H and fig. S22; CD45^+^CD3^+^ cells, 34.8% for NanoICD/BSA and 24.1% for PTX) than other treatments, indicating the ability of NanoICD to maintain cytotoxic T lymphocyte (CTL) proliferation/activity and elicit T cell–mediated antitumor immunity. Next, a standard vaccination assay was performed to determine whether NanoICD/BSA is sufficient to drive bona fide ICD and elicit protective cognate anticancer immunity against tumor recurrence and metastasis (*28*, *30*, *44*). B16F10 cells were first exposed to ETL, nBSA, and NanoICD/BSA and then washed and resuspended in PBS to remove treatment reagents. Cells succumbing to PTX were used as the positive control. The pretreated cells and PBS (no vaccination control) were then injected subcutaneously into the left flank of C57BL/6 mice. One week later, the mice were rechallenged by injecting live B16F10 into the contralateral flank (Fig. 4I) or vein (Fig. 4J). The tumor-free survival of the mice was continuously monitored for 60 days after the challenge. As summarized in Fig. 4I, inoculation of NanoICD/BSA- and PTX-treated cells conferred immunological protection against tumor recurrence in 40 and 20% of the mice. In addition, the inoculation of NanoICD/BSA- and PTX-treated cells also prevented lung metastasis of B16F10 cells (Fig. 4J). Collectively, these results indicate that NanoICD/BSA can effectively drive bona fide ICD and induce protective cognate anticancer immunity.

### Enhancement of antitumor immune responses by NanoICD/CAT

**Fig. 5.**
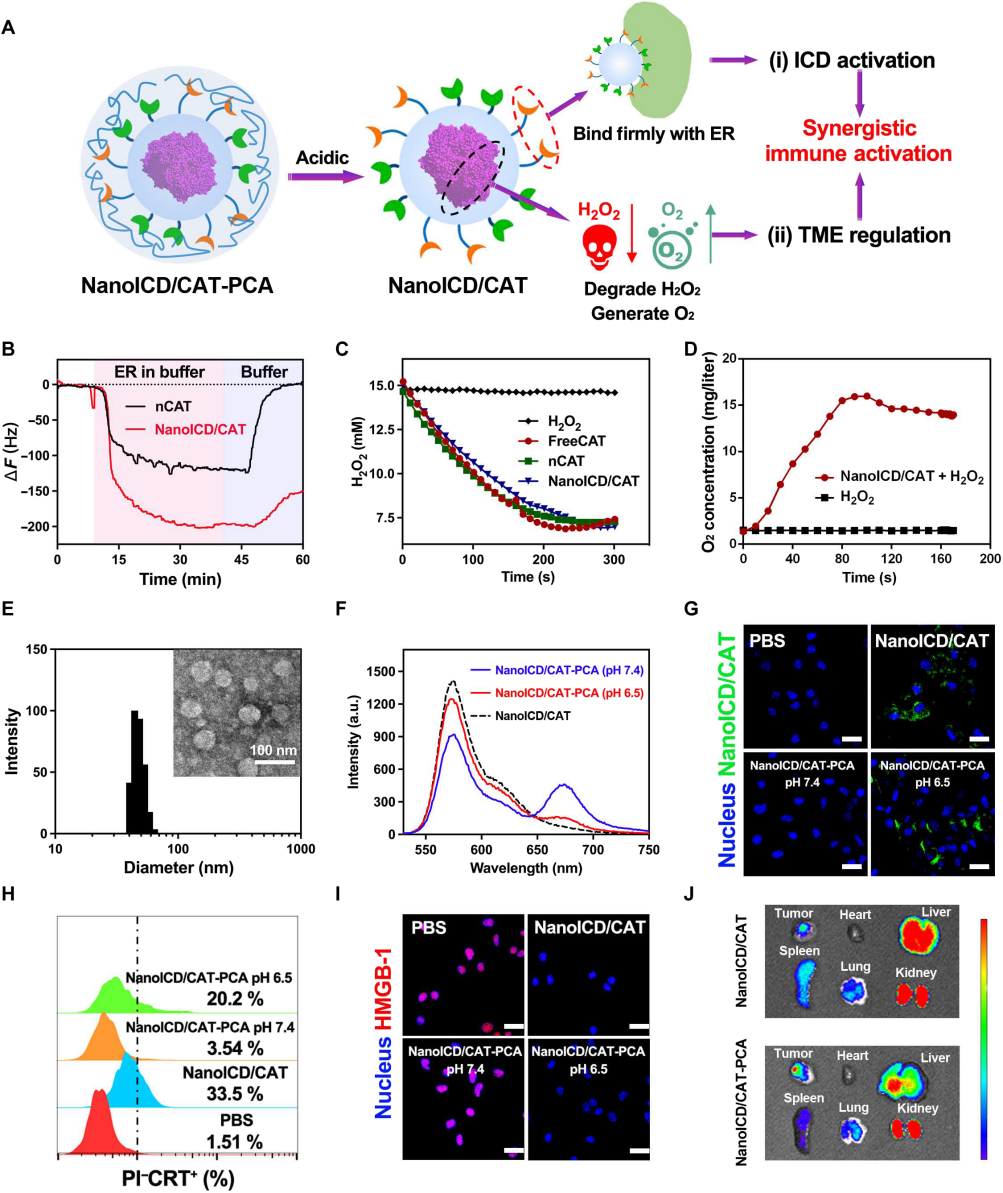
Schematic and the characterization of NanoICD/CAT-PCA. (**A**) Schematic illustration of NanoICD/CAT-PCA for synergistic immune activation. (**B**) QCM-D experiment showing the binding affinities between nCAT, NanoICD/CAT, and ER. (**C**) H_2_O_2_ degradation after treatment with CAT, nCAT, and NanoICD/CAT. (**D**) O_2_ generation after treatment with CAT, nCAT, and NanoICD/CAT. (**E**) Size distribution and TEM image of NanoICD/CAT-PCA. Scale bar, 100 nm. (**F**) Fluorescence spectra of NanoICD/CAT and NanoICD/CAT-PCA after incubation under different conditions. (**G**) Cellular uptake efficacy of NanoICD/CAT and NanoICD/CAT-PCA under different conditions. (**H**) Pre-apoptotic CRT exposure on cell surface after treatment with PBS, NanoICD/CAT, NanoICD/CAT-PCA (pH 7.4), and NanoICD/CAT-PCA (pH 6.5). (**I**) The exodus of HMGB-1 from cell nuclei after different treatments. (**J**) Ex vivo images of tumor and major organs of mice treated with NanoICD/CAT and NanoICD/CAT-PCA at 24 hours after injection. The CAT of NanoICD/CAT and NanoICD/CAT-PCA was prelabeled with Cy5. Scale bars, 25 μm. a.u., arbitrary units.

Despite the effective generation of ER stress and the activation of ICD by NanoICD/BSA, the therapeutic efficacy may still be far from expected because of the immunosuppressive environment of the tumor (*1*, *45*). Inflammation and hypoxia are the two most studied indicators of immunosuppression, which impair antitumor immune responses by reducing the migratory, cytolytic, and survival of effector T cells and promoting the infiltration of immunosuppressive cells and the accumulation of proinflammatory mediators (*46*). Therefore, simultaneous immune activation and inflammation/hypoxia alleviation may be a promising strategy to enhance the antitumor efficacy. Different from traditional ICD inducers, the highly customizable structure of NanoICD provides the potential to integrate immunomodulating functions by replacing the BSA with other functional proteins and enzymes during the synthesis. In addition, coating NanoICD with a TME-responsive polymer allows effective regulation of its biodistribution after systemic administration. CAT, which catalyzes the decomposition of H_2_O_2_ into O_2_, emerges as an ideal candidate for alleviating immunosuppressive inflammation and the hypoxic TME. For demonstration, CAT was used to perform the nanoencapsulation to synthesize NanoICD/CAT. Subsequently, NanoICD/CAT was coated with a poly(ethylene glycol) (PEG)–based pH-responsive polymer (PCA) to obtain NanoICD/CAT-PCA (Fig. 5A) (*14*, *16*). The PEG segments of the coating effectively protect NanoICD/CAT-PCA from clearance by the mononuclear phagocytosis system (MPS) and prevents undesired ICD activation. Upon reaching acidic TME, the polymer coating was dissociated, allowing the release and cellular uptake of NanoICD/CAT. Last, the surface interaction between NanoICD/CAT and ER leads to the activation of ICD-associated immune responses, while the integrated CAT efficiently alleviates tumor immunosuppression by decomposing H_2_O_2_ to O_2_, resulting in the activation and enhancement of anti-tumor immune responses.

To construct NanoICD/CAT-PCA, NanoICD/CAT was prepared using a similar nanoencapsulation strategy by replacing BSA with CAT (details in the Supplementary Materials). After the preparation, we first evaluated the mass of NanoICD/CAT bound to ER using QCM-D. Nanoparticles with structures similar to NanoICD/CAT but without ETL (referred to as nCAT) were synthesized as a comparison group. As shown in Fig. 5B, NanoICD/CAT (Δ*F* =198.95 Hz) exhibited a significantly higher maximum frequency shift than nCAT (Δ*F* = 124.05 Hz). When rinsed with PBS, only a slight change in frequency shift was observed in NanoICD/CAT (Δ*F* = 146.12 Hz, ΔΔ*F* = 52.83 Hz) compared to nCAT (Δ*F* = 1.79 Hz, ΔΔ*F* = 122.26 Hz), suggesting the strong binding of NanoICD/CAT to ER. Next, the catalytic activity of NanoICD/CAT was evaluated. As shown in fig. S23, 88.3% of the original CAT activities were retained after the nanoencapsulation. The catalytic activity enables NanoICD/CAT to decompose H_2_O_2_ (Fig. 5C) and generate O_2_ effectively (Fig. 5D), thus providing the potential to alleviate tumor hypoxia and remodel immunosuppressive TME to enhance cancer immunotherapy.

To avoid MPS clearance and undesired immune activation, NanoICD/CAT was coated with a PEG-based pH-responsive polymer, PCA (mPEG_113_-PLys_120_/CA, PCA), to obtain NanoICD/CAT-PCA (detailed preparation in the Supplementary Materials). Transmission electron microscopy (TEM) and dynamic light scattering indicated the successful formation of spherical nanoparticles with a diameter of 44.25 nm and a zeta potential of −9.44 mV (Fig. 5E and fig. S24). Förster resonance energy transfer (FRET)–based analysis shows that a significant FRET signal was observed from NanoICD/CAT-PCA (Fig. 5F), indicating the successful coating of the polymer on NanoICD/CAT. The polymer coating was readily detached from NanoICD/CAT-PCA upon reaching acidic TME, which was confirmed by the decrease in FRET signal when NanoICD/CAT-PCA was incubated at pH 6.5. In addition, the cellular uptake efficiency of NanoICD/CAT-PCA was investigated under different conditions. As shown in the confocal images (Fig. 5G), negligible fluorescence signal was detected from cells treated with NanoICD/CAT-PCA (pH 7.4). In contrast, significantly higher fluorescence signals were observed in cells treated with NanoICD/CAT and NanoICD/CAT-PCA (pH 6.5). This result was then confirmed by the flow cytometric analysis (fig. S25). The significantly different cellular uptake behaviors of NanoICD/CAT-PCA under different conditions (pH 7.4 and 6.5) may provide an opportunity to precisely control the activation of ICD-associated antitumor immunity. For demonstration, the ICD-inducing ability of NanoICD/CAT-PCA was then investigated under different conditions. Consistent with its cellular uptake behavior, NanoICD/CAT-PCA effectively induced CRT exposure (Fig. 5H and fig. S26), HMGB-1 release (Fig. 5I), and ATP secretion (fig. S27) only under acidic conditions (pH 6.5), but not under physiological conditions (pH 7.4). In addition, the polymer coating significantly improved the biodistribution of NanoICD/CAT, with significantly enhanced fluorescence signals observed in tumor tissues of mice treated with NanoICD/CAT-PCA compared to NanoICD/CAT (Fig. 5J). All these results indicate that NanoICD/CAT-PCA can be enriched and activated at the tumor site after systemic administration, which is important for enhancing the efficiency of immunotherapy and reducing side effects.

### NanoICD/CAT-PCA for enhanced cancer immunotherapy

**Fig. 6.**
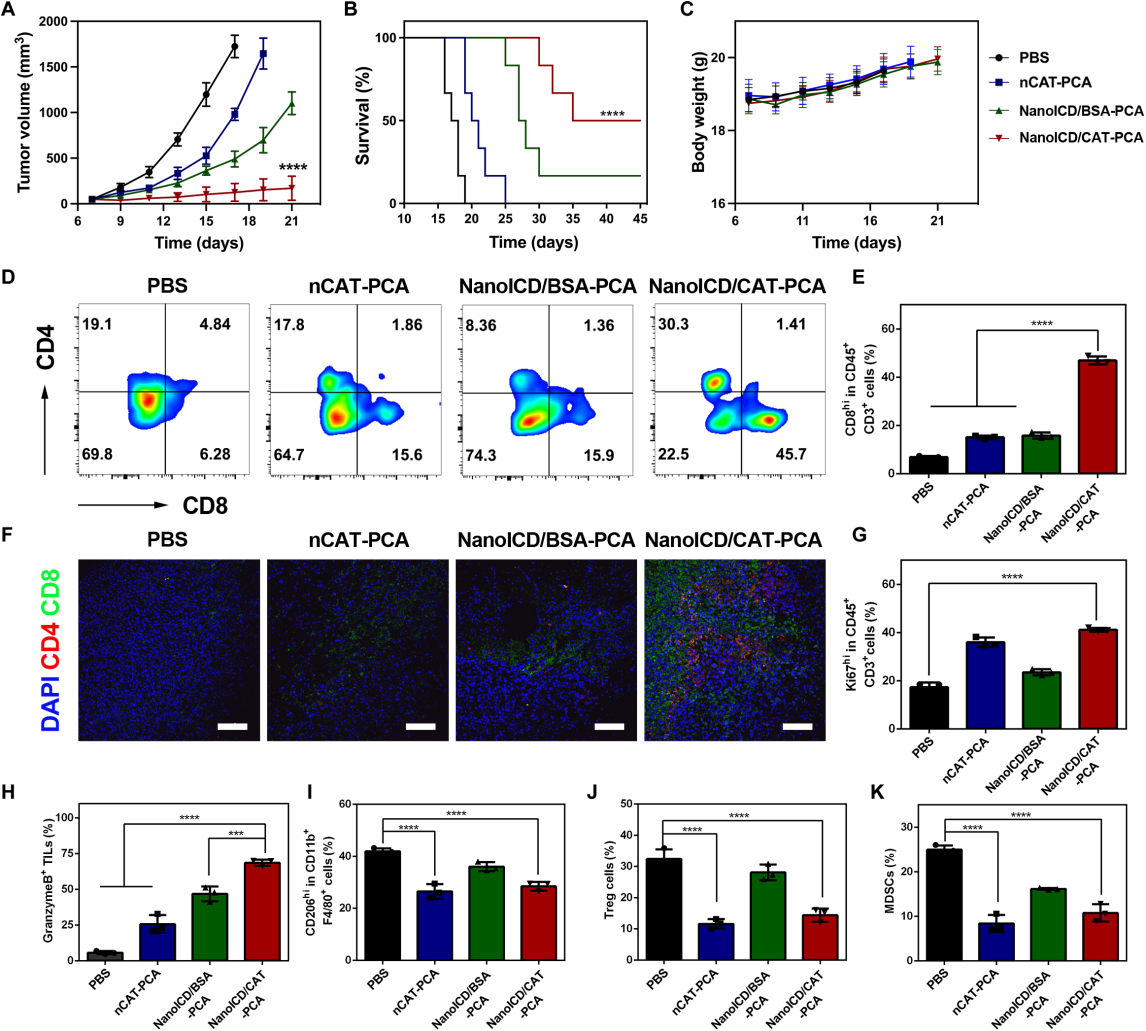
In vivo antitumor efficacy of NanoICD/CAT-PCA. (**A**) Average tumor growth kinetics in different groups (PBS, nCAT-PCA, NanoICD/BSA-PCA, and NanoICD-PCA), *n* = 6. (**B**) The survival of tumor-bearing mice after different treatments as indicated. (**C**) The variations of mouse weight during treatment, *n* = 6. (**D** and **E**) The population of CD4^+^ T cells and CD8^+^ T cells (gated on CD45^+^CD3^+^) within tumors of mice after receiving different treatments (D) and quantitative results (E). (**F**) Immunofluorescence images showing the infiltration of CD8^+^ T cells and CD4^+^ T cells within tumors after different treatments. (**G** to **K**) Quantitative analysis of Ki67^hi^CD8^+^ T cells (G), granzyme B^+^ CD8^+^ T cells (H), M2-like TAMs (I), T_reg_ cells (J), and MDSCs (K) in tumors from mice treated with PBS, nCAT-PCA, NanoICD/BSA-PCA, and NanoICD/CAT-PCA. Data are presented as means ± SD of six independent experiments for (A) to (C) (biological replicates, *n* = 6) and three independent experiments for (E) and (G) to (K) (biological replicates, *n* = 3). Significant levels are ***P < 0.001 and ****P < 0.0001. Scale bars, 100 μm.

Next, we evaluated the antitumor efficacy of NanoICD/CAT-PCA by simultaneously inducing ICD and remodeling the TME in vivo. To demonstrate the versatility and effectiveness of the NanoICD strategy in cancer immunotherapy, 4T1 breast tumor, which is another tumor model with lower immunogenicity, was used for in vivo studies. For better demonstration, PCA-coated nCAT (nCAT-PCA), which can only remodel immunosuppressive TME, and PCA-coated NanoICD/BSA (NanoICD/BSA-PCA), which can only induce ICD, were used as the comparison groups to perform the same test. As shown in Fig. 6A and fig. S28, NanoICD/CAT-PCA exhibited the most effective tumor suppression in mice compared to other treatments. As a result, NanoICD/CAT-PCA significantly improved the survival of 4T1-bearing BALB/c mice, with 50% of tumor-bearing mice alive at the observed 45 days (Fig. 6B). In addition, negligible body weight loss was observed during the treatment (Fig. 6C). Histopathological analysis of major organs, including heart, liver, spleen, lung, and kidney, showed no obvious abnormalities or damage after the treatment, indicating the safety of nCAT-PCA (fig. S29).

To investigate the mechanism underlying the enhanced anti-tumor effect, the tumor tissues were harvested for flow cytometric analysis. As shown in Fig. 6 (D and E), a significant increase in the infiltration of cytotoxic CD8^+^ T cells (CTLs, CD45^+^CD3^+^) was observed in the tumor tissues of NanoICD/CAT-PCA (45.7%) treatment compared to those of PBS (6.28%), nCAT-PCA (15.6%), and NanoICD/BSA-PCA (15.9%). CLSM observation of the tumor tissues confirmed these results (Fig. 6F), with the highest fluorescent signal of CD8^+^ CTLs observed in tumor sections from mice treated with NanoICD/CAT-PCA. Furthermore, significantly improved proliferation (Ki67^hi^CD8^+^ cells; Fig. 6G and fig. S30) and activation (granzyme B^+^ CD8^+^; Fig. 6H and fig. S31) of CTLs were also observed in the tumor of NanoICD/CAT-PCA–treated mice, indicating the effective activation of T cell–based antitumor immunity. In addition, the intratumoral proportions of immunosuppressive cells were also analyzed by flow cytometry. As shown in the results, nCAT-PCA and NanoICD/CAT-PCA treatments resulted in a significant reduction of M2-like tumor-associated macrophages (M2-TAMs) (Fig. 6I and fig. S32), regulatory T cells (T_regs_) (Fig. 6J and fig. S33), and myeloid-derived suppressor cells (MDSCs) (Fig. 6K and fig. S34), indicating the effective alleviation of immunosuppression by the catalytic activity of CAT. Further analysis of cytokine (interleukin-10, IL-10; fig. S35A) and immunosuppressive cytokines (transforming growth factor–β, TGF-β; fig. S35B) confirmed these results, with nCAT-PCA and NanoICD/CAT-PCA treatment showing a significant down-regulation of IL-10 and TGF-β.

**Fig. 7.**
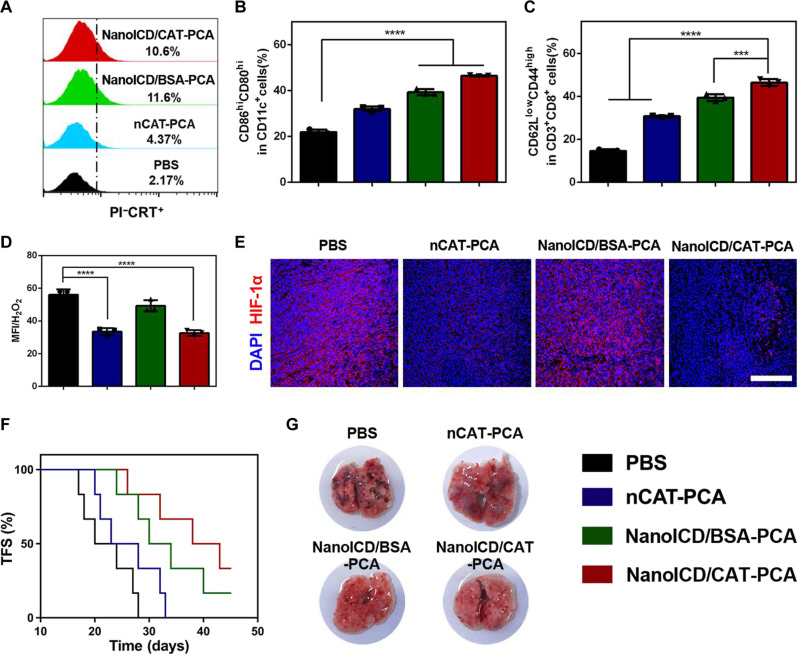
NanoICD/CAT-PCA activates ICD and remodels immunosuppressive TME in vivo. (**A**) Pre-apoptotic CRT exposure on tumors from mice treated with PBS, nCAT-PCA, NanoICD/BSA-PCA, and NanoICD/CAT-PCA. (**B**) The population of CD80^+^CD86^+^ DC cells (gated on CD11c^+^) in tumor-draining lymph nodes of mice after receiving different treatments. (**C**) The population of CD44^+^CD62L^−^ effector memory cells (gated on CD3^+^CD8^+^) in spleen of mice after receiving different treatments. (**D**) Flow cytometric analysis showing H_2_O_2_ concentrations in tumor tissues after different treatments. (**E**) Immunofluorescence images showing HIF-1α concentrations in tumor tissues after different treatments. (**F**) The TFS of 4T1-bearing BALB/c mice after different treatments as indicated. (**G**) The lung metastases in mice after the indicated treatments. Scale bar, 200 μm. Data are presented as means ± SD from three independent experiments for (B) to (D) (biological replicates, *n* = 3) and six independent experiments for (F) (biological replicates, *n* = 6). Significant levels are ***P < 0.001 and ****P < 0.0001. Scale bar, 200 μm.

The significantly improved antitumor immunity exhibited by NanoICD/CAT-PCA can be attributed to the synergy between ICD activation and the remodeling of the immunosuppressive TME. To this end, the surface CRT exposure in tumor cells was first analyzed using flow cytometry (fig. S36). As shown in Fig. 7A, significantly higher PI^−^CRT^+^ cell population was observed from the tumor sections of mice treated with NanoICD/BSA-PCA and NanoICD/CAT-PCA, which is consistent with the results obtained from immunofluorescence imaging. Furthermore, elevated levels of mature DCs (Fig. 7B and fig. S37) and effector memory T cells (Fig. 7C and fig. S38) were identified in tumors of mice treated with NanoICD/BSA-PCA and NanoICD/CAT-PCA, indicating the activation of ICD-associated antitumor immune responses. Next, we assess the ability of NanoICD/CAT-PCA to mitigate the inflammation and the hypoxia of TME. The H_2_O_2_ concentrations in tumors were measured through the utilization of an OxiVisionGreen probe. We found that both NanoICD/CAT-PCA and nCAT-PCA treatments significantly reduced fluorescence signals (Fig. 7D) of OxiVisionGreen, indicating a decrease in H_2_O_2_ concentration within tumors. This suggests that inflammation within the TME was effectively alleviated. In addition, the decomposition of H_2_O_2_ led to the generation of O_2_ and consequent alleviation of hypoxic conditions of TME, which was confirmed by the down-regulation of hypoxia-inducible factor–1α (HIF-1α, Fig. 7E). As a result of these properties, NanoICD/CAT-PCA efficiently elicited protective cognate antitumor immunity against tumor rechallenge (Fig. 7F) and tumor lung metastasis (Fig. 7G). These findings highlight the great potential of NanoICD as a platform technology for enhanced cancer immunotherapy.

## DISCUSSION

Nanoparticle-based cancer immunotherapy has shown promising therapeutic potential in clinical settings. However, current research mainly focuses on the use of nanoparticles as delivery vehicles but often overlooks their potential to directly modulate immune responses. Although some serendipitous discoveries suggest that certain nanoparticles may have immunoregulatory effects, there is a lack of rationally designed nanoparticles with immunomodulatory capacity. To this end, we have presented a novel synthetic nanoparticle, NanoICD, capable of inducing ICD. NanoICD has a rationally designed surface containing two types of functional groups that determine its surface charge and ER-targeting potential. By controlling monomer composition and formulation methods, NanoICD achieves efficient accumulation in the ER, induces ER stress, and ultimately activates ICD-associated immune responses (Figs. 2 and 3). Animal studies based on NanoICD/BSA confirmed the immunomodulatory function of the polymer surface (Fig. 4). Furthermore, the synthesis method of NanoICD is generally applicable to various proteins and enzymes, so that the biological functions of the encapsulated protein or enzyme can synergize with the ICD-inducing function of the polymer surface to achieve enhanced antitumor performance (Fig. 5). As demonstrated by encapsulating CAT, NanoICD/CAT-PCA effectively alleviated the immunosuppressive TME and induced robust antitumor immune responses in 4T1 tumor–bearing mice (Fig. 6), significantly suppressing tumor recurrence and lung metastasis, and improving the survival rate of the mice (Fig. 7). These unique features make NanoICD a novel strategy for effective cancer immunotherapy.

Moreover, the research presented in this study illustrates how a specifically engineered nanoparticle is capable of modulating the immune system independent of conventional immunomodulatory drugs. The study on the structure-activity relationship confirmed the critical role of surface properties in the ICD-inducing ability of NanoICD. As the design of NanoICD was inspired by the endogenous ER stress generated by unfolding/misfolding proteins, this work demonstrates the potential of artificial nanostructure with a tailor-made surface to regulate biological processes autonomously. Consequently, this study provides a novel perspective for the development of advanced nanomedicines for cancer immunotherapy.

Despite the significant efficacy of NanoICD in activating ICD-associated antitumor immune response, there remain certain issues that necessitate our attention and resolution in future studies. (i) The impact of macrophage phagocytosis on the overall ICD process. Macrophage phagocytosis of NanoICD diminishes its bioavailability in tumor cells and may lead to unpredictable alterations in these macrophages. Thus, improving the targeting and selectivity of NanoICD for tumor cells is imperative in our ongoing research. (ii) The role of don’t-eat-me signals. While NanoICD effectively induce the exposure of CRT on tumor surface and prompt the endocytosis of tumors by phagocytic cells, its efficacy may still be limited by don’t-eat-me signals, such as CD47. Therefore, the combination of NanoICD with inhibitors targeting the don’t-eat-me signals, such as anti-CD47, is expected to yield broader clinical benefits. (iii) The variability of human cell lines compared to B16F10 and 4T1. Although we have demonstrated in human cancer cell lines that NanoICD induces the release of DAMPs, including CRT, HMGB-1, and ATP (fig. S39), it is imperative to conduct further investigations, particularly in patient-derived xenografts and humanized mouse models, to corroborate and validate this observation.

## MATERIALS AND METHODS

### Materials

All commercially available reagents and solvents were used as received without further purification unless otherwise noted. BSA and CAT were purchased from Mreda. AAm and *N*-acryloxysuccinimide (NAS) were purchased from Aladdin. APm and *N*-tosylethylenediamine were obtained from Energy Chemical. Acryloyl chloride, ammonium persulfate (APS), BIS, and *N,N,N′,N′*-tetramethylethylenediamine (TEMED) were purchased from Bidepharm. Antibodies for CLSM observation and flow cytometry were purchased from BioLegend unless otherwise noted: phycoerythrin (PE)–conjugated anti-mouse CD11b (dilution 1:200, catalog no. 101207), fluorescein isothiocyanate (FITC)–conjugated anti-mouse CD80 (dilution 1:200, catalog no. 104705), allophycocyanin (APC)–conjugated anti-mouse CD86 (dilution 1:200, catalog no. 105011), APC-conjugated anti-mouse CD11c (dilution 1:200, catalog no. 117309), FITC-conjugated anti-mouse SIINFEKL-MHC I (dilution 1:200, catalog no. 116505), APC-conjugated anti-mouse CD3 (dilution 1:200, catalog no. 100235), PE-conjugated anti-mouse CD8a (dilution 1:200, catalog no. 110707), FITC-conjugated anti-mouse CD44 (dilution 1:200, catalog no. 156007), APC/Cy7-conjugated anti-mouse CD62L (dilution 1:200, catalog no. 104427), APC/Cy7-conjugated anti-mouse CD45 (dilution 1:200, catalog no. 103115), FITC-conjugated anti-mouse Ki-67 (dilution 1:200, catalog no. 652409), FITC-conjugated anti-mouse granzyme B (dilution 1:200, catalog no. 515403), APC-conjugated anti-mouse F4/80 (dilution 1:200, catalog no. 123115), PerCP/Cy5.5-conjugated anti-mouse CD206 (dilution 1:200, catalog no. 141715), APC-conjugated anti-mouse CD25 (dilution 1:200, catalog no. 101910), PE-conjugated anti-mouse FOXP3 (dilution 1:200, catalog no. 118903), FITC-conjugated anti-mouse Gr-1 (dilution 1:200, catalog no. 108405), and FITC-conjugated anti-mouse CD4 (dilution 1:200, catalog no. 100405). ATTO 488–conjugated anti-human CRT (dilution 1:200, catalog no: SPC-122) was obtained from Stressmarq. ELISA kits for HMGB-1 (E-EL-M0676c) and IL-10 (E-EL-M0046c) were purchased from Elabscience Biotechnology Co. Ltd., and TGF-β was purchased from Tianjin Anorikang Biotechnology Co. Ltd. Antibodies for Western blot were purchased from Abcam. AV-FITC apoptosis detection kit, ATP assay kit, paraformaldehyde, ER-Tracker Red, and Triton X-100 were purchased from Beijing Solarbio Science & Technology Co. Ltd. Lysosome-Tracker Red was obtained from Beyotime. OxiVision Green hydrogen peroxide sensor was purchased from AAT Bioquest.

### Instruments

Nuclear magnetic resonance data were recorded on a Bruker AV400 spectrometer. Ultraviolet-visible spectra were recorded with a Nanodrop One^c^ (Thermo Fisher Scientific, USA). Size distribution and zeta potential were performed on a Brookhaven ZETAPALS/BI-200SM (Brookhaven Instrument, USA). TEM measurements were performed on a Talos F200C electron microscope at an acceleration voltage of 120 kV. The mass spectrum of ETL was performed on a Fouier transform ion cyclotron resonance mass spectrometer (Varian 7.0T FTMS). Flow cytometry analysis was performed on a BD LSR Fortessa flow cytometry. CLSM images were captured on a FluoView Confocal Laser Scanning Microscopes-FV1000. Living imaging was performed with an IVIS Lumina imaging system (Caliper Life Sciences, USA). Tumor tissues, spleen, and tumor-draining lymph nodes were homogenized with the GentleMACs Dissociator (FJ200-SH). The binding affinities between nanoparticles and ER were analyzed with QCM-D, equipped with a Q-Sence E4 system (Q-Sence, Västra Frölunda, Sweden).

### Syntheses of ETL

To a solution of *N*-tosylethylenediamine (1.35 g, 6.3 mmol) in CH_2_Cl_2_ (20 ml), acryloyl chloride (0.855 g, 9.45 mmol) and triethylamine (0.96 g, 9.45 mmol) in CH_2_Cl_2_ (10 ml) were added dropwise to the solution at 0°C and stirred at room temperature for another 2 hours. The reaction mixtures were then extracted twice with water and saturated sodium chloride to remove unreacted acryloyl chloride and triethylamine. The prepared ETL was then purified by column chromatography (PE:EA = 1:1) (yield, 1.61 g, 95%).

### Preparation of NanoICD

NanoICD was synthesized by a polymerization method that forms a thin layer of cross-linked polymer around each protein molecule. Before polymerization, proteins (BSA or CAT) were reacted with NAS to attach polymerization sites onto the surface, which was achieved by adding NAS [10% in dimethyl sulfoxide (DMSO) w/v] to the protein solution (5 mg/ml, pH 8.5, 50 mM sodium carbonate buffer) at a molar ratio of 20:1 (NAS/protein) and reacting for 1 hour at 4°C. The solution was then thoroughly dialyzed against pH 8.5 sodium carbonate buffer (50 mM) using a dialysis bag [molecular weight cutoff (MWCO) = 10 kDa]. Monomers and cross-linkers, which were first prepared as stock solutions [AAm and APm: 20% (w/v) in deionized water, ETL, and BIS: 10% (w/v) in DMSO], were then added to the protein solution. The final protein concentration was adjusted to 1 mg/ml by dilution with sodium carbonate buffer (50 mM, pH 8.5). The polymerization was initiated by the addition of TEMED/APS (2:1, w/w) and kept at 4°C for 1 hour. After the polymerization, the unreacted monomers and cross-linkers were removed by centrifugal filtration (MWCO = 10 kDa). To prepare NanoICD/BSA with different zeta potentials, the SA was used to neutralize the polymerized APm in NanoICD/BSA to varying degrees. In detail, SA was first prepared as a stock solution (10 mg/kg in DMSO), then added to the NanoICD/BSA solution (5 ml, 1 mg/ml, pH 8.5, 50 mM sodium carbonate buffer) at different molar ratios (SA/BSA, 25:1, 50:1, and 100:1), and reacted for 1 hour at 4°C. The unreacted SA was then removed by centrifugal filtration (MWCO = 10 kDa). The final protein concentration was diluted to 1 mg/ml with sodium carbonate buffer and stored at 4°C for further use. The molar ratios for the preparation of different NanoICD are provided in tables S1 and S2.

### Preparation of NanoICD/CAT-PCA

To prepare NanoICD/CAT-PCA, a PEG-based pH-responsive polymer, PCA, was first synthesized according to our previously reported methods (*47*, *48*). Next, PCA was dissolved in sodium carbonate buffer (pH 8.5, 50 mM) to make a 10 mg/ml stock solution and mixed with NanoICD/CAT at a 10:1 molar ratio (PCA/BSA) to afford NanoICD/CAT-PCA.

### Investigation of the mass of NanoICD bound to ER

The mass of NanoICD bound to ER was measured using a QCM-D. Au sensor chips were first modified with nBSA, NanoICD/BSA-20, NanoICD/BSA-40, nCAT, and NanoICD/CAT (1.5 μM) and then placed in the standard flow module (detailed in the Supplementary Materials). Each sensor chip was washed with PBS buffer for 1 hour at a flow rate of 10 μl/min and then equilibrated at a flow rate of 2 μl/min until the baseline was stable. Then, freshly extracted ER was added to the flow buffer and allowed to run at the flow rate of 2 μl/min for 30 min. After reaching equilibrium, the flow phase was replaced with PBS (flow rate of 2 μl/min). All the QCM experiments were performed at 37°C.

### Vaccination assays

All animal studies were performed in accordance with the Regulations for the Administration of Affairs Concerning Experimental Animals (Tianjin, revised in June 2004) and were approved by the Animal Ethics Committee of Nankai University (approval number, 2023-SYDWLL-000235). The ability of NanoICD/BSA to prevent tumor recurrence was evaluated using a vaccine assay. Briefly, 1 × 10^6^ B16F10 cells were first incubated with ETL (100 μM), PTX (15 μM), nBSA (1.5 μM), and NanoICD/BSA (1.5 μM) for 24 hours and then washed and resuspended in PBS. Subsequently, the treated cells and PBS (no vaccination control) were inoculated subcutaneously into the lower flank of 6-week-old female C57BL/6 mice (vaccination). One week later, the mice were subcutaneously injected with 1 × 10^5^ untreated B16F10 cells into the contralateral flank (rechallenge). Tumor incidence was routinely monitored up to 60 days after the rechallenge.

The ability of NanoICD/CAT-PCA to prevent tumor recurrence was evaluated with a similar vaccine assay using a different type of cancer cells. Briefly, 1 × 10^6^ 4T1 cells were inoculated subcutaneously into the left mammary fat pad of 6-week-old female BALB/c mice. One week later, 200 μl of PBS, nCAT-PCA (4 μM, 10 mg/kg), NanoICD/BSA-PCA (4 μM, 2.66 mg/kg), and NanoICD/CAT-PCA (4 μM, 10 mg/kg) were injected intravenously every 2 days for a total of three doses. Subsequently, 1 × 10^5^ 4T1 cells were inoculated subcutaneously into the contralateral flank (rechallenge). Tumor incidence was routinely monitored up to 45 days after the rechallenge.

### Statistical analysis

Data are presented as means ± SD from at least three independent experiments (*n* ≥ 3), and the significance levels are *P < 0.05, **P < 0.01, ***P < 0.001, and ****P < 0.0001, analyzed by one-way or two-way analysis of variance (ANOVA) (when more than two groups were compared) with a Dunnett’s test. *P* < 0.05 or less was considered significant.
