# Supplementary material for: Rational design of ICD-inducing nanoparticles for cancer immunotherapy
Source: Sci Adv. 2024 Feb 7;10(6):eadk0716. doi: 10.1126/sciadv.adk0716 (PMC10849581; doi:10.1126/sciadv.adk0716)
Supplement: Supplementary file 1 — Supplementary Text Figs. S1 to S39 Tables S1 to S4 [file sciadv.adk0716_sm.pdf]

Science Advances
AAAS

Supplementary Materials for

Rational design of ICD-inducing nanoparticles for cancer immunotherapy

Zhanzhan Zhang *et al.*

Corresponding author: Yang Liu, yliu@nankai.edu.cn

Sci. Adv. **10**, eadk0716 (2024)
DOI: 10.1126/sciadv.adk0716

This PDF file includes:

Supplementary Text
Figs. S1 to S39
Tables S1 to S4

Supplementary Text

Cell culture

The mouse melanoma cells B16F10 and mouse breast cell 4T1 were purchased from the American Type Culture Collection (ATCC) and maintained in Dulbecco's modified Eagle's medium (DMEM) with 10 % fetal bovine serum (FBS, v/v), 100 U/ml penicillin and streptomycin in a humidified atmosphere of 5 % CO_2 at 37 ˚C.

Syntheses of ETL

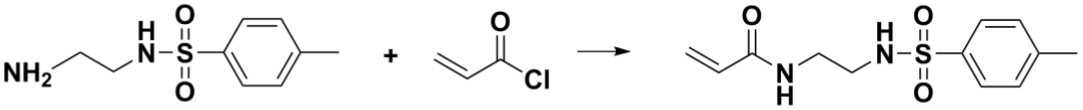

^{1}H NMR (400 MHz, DMSO-d_6, δ): 8.16 (t, J = 5.9 Hz, 1H, -CO-*NH*-CH_2), 7.69 (t, 1H, -CH_2-*NH*-SO_2-), 7.67 (d, J = 7.9 Hz, 2H, Ar-H), 7.41 (d, J = 7.9 Hz, 2H, Ar-H), 6.17 (dd, J = 17.1, 10.0 Hz, 1H, CH_2=*CH*-), 6.06 (dd, J = 17.1, 2.4 Hz, 1H, C$H^1$$H^2$=CH-), 5.58 (dd, J = 9.9, 2.4 Hz, 1H, C$H^1$$H^2$=CH-), 3.17 (q, J = 6.5 Hz, 2H, -SO_2-NH-CH_2-), 2.78 (q, J = 6.6 Hz, 2H, -CO-NH-CH_2-), 2.39 (s, 3H,-CH_3) ppm.

^{13}C NMR (400 MHz, DMSO-d_6, δ): 166.56, 143.72, 136.57, 130.58, 129.91, 127.09, 126.96, 43.05, 39.47, 21.58 ppm.

MS:[M+Cl]: m/z: calcd. for ($C_{12}H_{16}N_2O_3SCl$):303.06, found 303.0573.

Investigation of the cellular uptake efficacy of NanoICD/BSA

In this study, B16F10 cells were seeded in 12-well plates at a density of 1 × 105 cells/well for a day prior exposure to FITC-labeled nBSA, NanoICD/BSA-*n* (*n* = 10, 20, 30, 40), and (*Z*)-NanoICD/BSA-40 (*Z*= +6, +3, +1.5, 0, -2 mV). After incubation for 2 h, the cells were collected and re-suspended in cold PBS for flow cytometry analysis. The concentration of all nanoparticles used in this study was 1.5 μM, calculated based on the concentration of the BSA.

Investigation of the ER-targeting and retention capability of NanoICD/BSA-*n*

CLSM-based analysis was conducted to investigate the ER-targeting and retention capability of NanoICD/BSA-*n*. Briefly, B16F10 cells were seeded in a 35 mm confocal dish (Φ =15 mm) at a density of 2 × 10^4 cells/well for a day prior exposure to FITC-labeled nBSA (1.5 μM), NanoICD/BSA-10 (1.5 μM), NanoICD/BSA-20 (1.5 μM), NanoICD/BSA-30 (1.5 μM), and NanoICD/BSA-40 (1.5 μM). After incubation for a certain period (2 h, 4 h, 6 h, or 8 h), the cells were rinsed with PBS and incubated with ER-Tracker Red at 37 ˚C for 30 min according to the manufacturer's instructions. Subsequently, the cells were fixed in 4 % paraformaldehyde for 15 min. Finally, the cells were stained with 4, 6-diamidino-2-phenylindole dihydrochloride (DAPI) for CLSM analysis, and the co-localization of the NanoICD/BSA-n with ER was quantitatively assessed by analyzing the Manders' coefficients M2 (fraction of NanoICD overlapping ER) using ImageJ-JACoP.

Study of the endosome escape capability of NanoICD/BSA

In this study, B16F10 cells were seeded in a 35 mm confocal dish (Φ =15 mm) at a density of 2×10^4 cells/well for a day prior exposure to FITC-labeled nBSA (1.5 μM), NanoICD/BSA-10 (1.5 μM), NanoICD/BSA-20 (1.5 μM), NanoICD/BSA-30 (1.5 μM), and NanoICD/BSA-40 (1.5 μM). After incubation for 2 h, the cells were rinsed with PBS and incubated with Lysosome-Tracker Red at 37 ˚C for 30 min following the manufacturer's instructions. Subsequently, the cells were fixed in 4 % paraformaldehyde for 15 min. Finally, the cells were stained with DAPI for CLSM analysis.

Investigation of the mass of NanoICD bound to ER

The mass of NanoICD bound to ER were measured using a QCM-D. Briefly, the Au sensor chips were first activated by immersing them in a solution of deionized water: ammonia water: 30% hydrogen peroxide = 5 : 1 : 1 (v/v/v) in an ice-water bath for 10 minutes. Following activation, the chips were rinsed with deionized water and dried using nitrogen gas. Next, the chips were modified with nanoparticles by immersing them in solutions of nBSA, NanoICD/BSA-20, NanoICD/BSA-40, nCAT, or NanoICD/CAT (1.5 μM) for 24 hours at room temperature in the presence of 2-imidothiolane hydrochloride (Traut's Reagent, 24 μM) and tris(2-carboxyethyl)phosphate hydrochloride (TCEP, 48 μM). Subsequently, the chips were rinsed with deionized water, dried with nitrogen gas, and put into the standard flow module. Each sensor chip was washed with PBS buffer for 1 h at a 10 μL/min flow rate, and then equilibrated at 2 μL/min until the baseline was stable. Then, freshly extracted ER (according to the manufacturer's instructions) in the flow buffer was injected for 30 min at 2 μL/min. After the binding reached equilibrium, the flow phase was replaced with PBS (flow rate 2 μL/min) to simulate the retro-translocation process of the ER. All of the QCM experiments in this study were operated at 37 °C.

Detection of calreticulin (CRT)

Flow cytometric measurement and immunofluorescence staining were used to detect proapoptotic CRT exposure on cell surfaces after different treatments. For flow cytometric-based analysis, B16F10 cells were seeded in 12-well plates at a density of 1×10^5 cells/well for a day prior exposure to PBS, PTX (15 μM), ETL (100 μM), nBSA (1.5 μM), NanoICD/BSA-*n* (*n* = 10, 20, 30, 40; 1.5 μM), (*Z*)-NanoICD/BSA-40 (*Z*= +6, +3, +1.5, 0, -2 mV; 1.5 μM), NanoICD/CAT (1.5 μM), NanoICD/CAT-PCA (1.5 μM, pH 7.4), and NanoICD/CAT-PCA (1.5 μM, pH 6.5). After incubation for 12 h, the cells were rinsed with PBS and incubated with ATTO488-conjugated antibody and propidium iodide (PI) according to the manufacturer's instructions for flow cytometry analysis.

For immunofluorescence imaging, B16F10 cells were seeded in a 35 mm confocal dish (Φ =15 mm) at a density of 2×10^4 cells/well for a day prior exposure to PTX (15 μM), ETL (100 μM), nBSA (1.5 μM), NanoICD/BSA (1.5 μM), NanoICD/CAT (1.5 μM), NanoICD/CAT-PCA (1.5 μM, pH 7.4), and NanoICD/CAT-PCA (1.5 μM, pH 6.5). After incubation for 12 h, the cells were rinsed with PBS and fixed with 4% paraformaldehyde at room temperature for 15 min. Nonspecific binding sites were blocked by pre-incubation with 5 % FBS in PBS for 30 min, followed by incubation with primary antibody for 1 h, and then incubated with the Alexa488-conjugated monoclonal secondary antibody for 30 minutes after three washes with PBS. Finally, the cells were stained with DAPI and examined by CLSM.

Distribution of intracellular high mobility group box 1 (HMGB-1)

Immunofluorescence imaging was used to study the distribution of intracellular HMGB-1 after different treatments. Briefly, B16F10 cells were seeded in 35 mm confocal dish (Φ =15 mm) at a density of 2 × 10^4 cells/well for a day prior exposure to PBS, PTX (15 μM), ETL (100 μM), nBSA (1.5 μM), and NanoICD/BSA (1.5 μM). After incubation for 12 h, the cells were rinsed with cold PBS, fixed with 4% paraformaldehyde at room temperature for 15 min, and permeabilized with 0.1 % Triton X-100 for 10 min. Nonspecific binding sites were blocked by pre-incubation with 5 % FBS in PBS for 30 min, followed by incubation with the primary antibody for 1 h, and then incubated with the Alexa594-conjugated monoclonal secondary antibody for 30 minutes after three washes with PBS. Finally, the cells were stained with DAPI for CLSM analysis. The extracellular content of HMGB-1 was evaluated by an HMGB-1 ELISA kit according to the manufacturer's instructions.

Secretion of ATP

The ATP secretion levels of the cells after different treatments were measured using a commercially available ATP assay kit. Briefly, B16F10 cells were seeded in 12-well plates at a density of 1 × 10^5 cells/well for a day prior exposure to PBS, PTX (15 μM), ETL (100 μM), nBSA (1.5 μM), and NanoICD/BSA (1.5 μM). After incubation for 12 h, the supernatant of the cell culture was collected, and the ATP content was measured using an ATP assay kit following the manufacturer's instructions.

Analysis of the signal pathway that induce ICD

Flow cytometric measurement and western blot-based analysis were employed to investigate the signal pathway that NanoICD/BSA induces ICD. For flow cytometry, B16F10 cells were seeded in 12-well plates at a density of 1 × 10^5 cells/well for a day prior exposure to PBS, PTX (15 μM), ETL (100 μM), nBSA (1.5 μM), and NanoICD/BSA (1.5 μM). After incubation for 12 h, the cells were rinsed with cold PBS, fixed with 4% paraformaldehyde at room temperature for 15 min, and permeabilized with 0.1 % Triton X-100 for 10 min. Nonspecific binding sites were blocked by pre-incubation with 5 % FBS in PBS for 30 min, followed by incubation with the primary antibody for 1 h, and then incubated with the Alexa488-conjugated monoclonal secondary antibody for 30 minutes after three washes with PBS. Finally, the cells were stained with DAPI for CLSM analysis. For western blot-based analysis, B16F10 cells were seeded in 6-well plates at a density of 2 × 10^5 cells/well overnight and then treated with PBS, PTX (15 μM), ETL (100 μM), nBSA (1.5 μM), and NanoICD/BSA (1.5 μM) for 24 h. After incubation, cells were rinsed with PBS and solubilized in 1% Nonidet P-40 lysis buffer. Homogenates were clarified by centrifugation at 20000g for 15 min at 4 ˚C, and protein concentrations were determined with a BCA assay. Total protein lysates were separated by SDS-PAGE on 10 % SDS acrylamide gels, which were then transferred to PVDF membranes (Millipore, USA). The membranes were incubated with primary antibodies against EIF2α, pEIF2α, and β-Actin (1:1000 dilution) overnight, followed by incubating with an HRP-conjugated secondary antibody (1:2000 dilution) for 1 h.

BMDC-mediated phagocytosis of cancer cells

In this study, bone marrow-derived dendritic cells (BMDCs) were first generated from the bone marrow of 8-week-old BALB/c mice. Next, B16F10 cells were stained with Calcein, AM (FITC channel) and then seeded in 12-well plates at a density of 1 × 10^5 cells/well for a day prior exposure to PBS, PTX (15 μM), ETL (100 μM), nBSA (1.5 μM), and NanoICD/BSA (1.5 μM). After incubation for 12 h, B16F10 cells were collected and then co-cultured with 1 × 10^6 BMDCs (pre-

stained with Dil, APC channel) for another 4 h. The BMDCs-mediated phagocytosis of cancer cells ($FITC^+APC^+$) was examined using flow cytometry measurement.

Antigen cross presentation and APC maturation

In this study, ovalbumin (OVA)-transfected B16F10 cells (B16F10-OVA) was employed as the cancer cell model. B16F10-OVA cells were first seeded in 12-well plates at a density of 1×10^5 cells/well overnight and then treated with PBS, PTX (15 μM), ETL (100 μM), nBSA (1.5 μM), and NanoICD/BSA (1.5 μM) for 12 h. After incubation, B16F10-OVA cells were collected and co-cultured with 1×10^6 BMDCs for another 48 h. Finally, BMDCs were stained with anti-SIINFEKL-MHCI-PE and anti-CD11c-APC for antigen cross presentation detection, or anti-CD11c-APC, anti-CD86-FITC and anti-CD80-PE for APC maturation detection.

Anti-tumor analysis

The female C57BL/6 mice and Balb/C mice at 6-8 weeks were purchased from Vital River Laboratory Animal Technology (Beijing, China). The anti-tumor efficacy of NanoICD/BSA was evaluated in a B16F10-bearing mice model. Briefly, 1×10^6 B16F10 cells were inoculated subcutaneously into the lower flank of 6-week-old female C57BL/6 mice. One week later, 200 μL of PBS, PTX (15 μM), ETL (100 μM), nBSA (1.5 μM), and NanoICD/BSA (1.5 μM) were injected intratumorally every 3 days for a total of 3 doses. The mice were sacrificed on 22 days post-treatment, and the tumors, tumor-draining lymph nodes (TDLNs), and spleen were collected for flow cytometric analysis.

The anti-tumor efficacy of NanoICD/CAT-PCA was evaluated in a 4T1-bearing mice model. Briefly, 1×10^6 4T1 cells were inoculated subcutaneously into the left mammary fat pad of 6-week-old female Balb/C mice. One week later, 200 μL of PBS, nCAT-PCA (4 μM, 10 mg/kg), NanoICD/BSA-PCA (4 μM, 2.66 mg/kg), and NanoICD/CAT-PCA (4 μM, 10 mg/kg) were injected intravenously every 2 days for a total of 5 doses. The mice were sacrificed on 22 days post-treatment, and the tumors, TDLNs, and spleen were collected for immunofluorescence staining and flow cytometric analysis.

Anti-metastasis assays

The ability of NanoICD/BSA to prevent tumor metastasis was evaluated using a vaccine assay. Briefly, 1×10^6 B16F10 cells were first incubated with ETL (100 μM), PTX (15 μM), nBSA (1.5 μM), and NanoICD/BSA (1.5 μM) for 24 h, then washed and resuspended in PBS. Subsequently, the treated cells and PBS (no vaccination control) were inoculated subcutaneously into the lower flank of 6-week-old female C57BL/6 mice (vaccination). One week later, the mice were intravenously injected with 1×10^5 untreated B16F10 cells. The mice were sacrificed on 10 days post-treatment, the lung was collected to evaluate the anti-metastasis ability of NanoICD/BSA.

The ability of NanoICD/CAT-PCA to prevent tumor recurrence was evaluated with a similar vaccine assay using a different type of cancer cells. Briefly, 1×10^6 4T1 cells were inoculated subcutaneously into the left mammary fat pad of 6-week-old female BALB/c mice. One week later, 200 μL of PBS, nCAT-PCA (4 μM, 10 mg/kg), NanoICD/BSA-PCA (4 μM, 2.66 mg/kg), and NanoICD/CAT-PCA (4 μM, 10 mg/kg) were injected intravenously every 2 days for a total of 3 doses. Subsequently, the mice were intravenously injected with 1×10^5 untreated B16F10 cells. The mice were sacrificed on 10 days post-treatment, the lung was collected to evaluate the anti-metastasis ability of NanoICD/CAT-PCA.

Flow cytometry analysis

Freshly harvested TDLNs, spleen, and tumor tissues were minced and homogenized using a GentleMACs Dissociator, and then passed through a 70×10^{-6} m cell strainer to obtain single-cell suspensions. The collected cells were diluted to 1×10^{7} cells/mL, and 100 μL of cells were stained with a cocktail of fluorescently conjugated antibodies. For intracellular staining, cells were first permeabilized with 100 μL fixation/permeabilization buffer before adding the antibody cocktail. After staining, the cells were fixed with 4 % paraformaldehyde and analyzed using a flow cytometer.

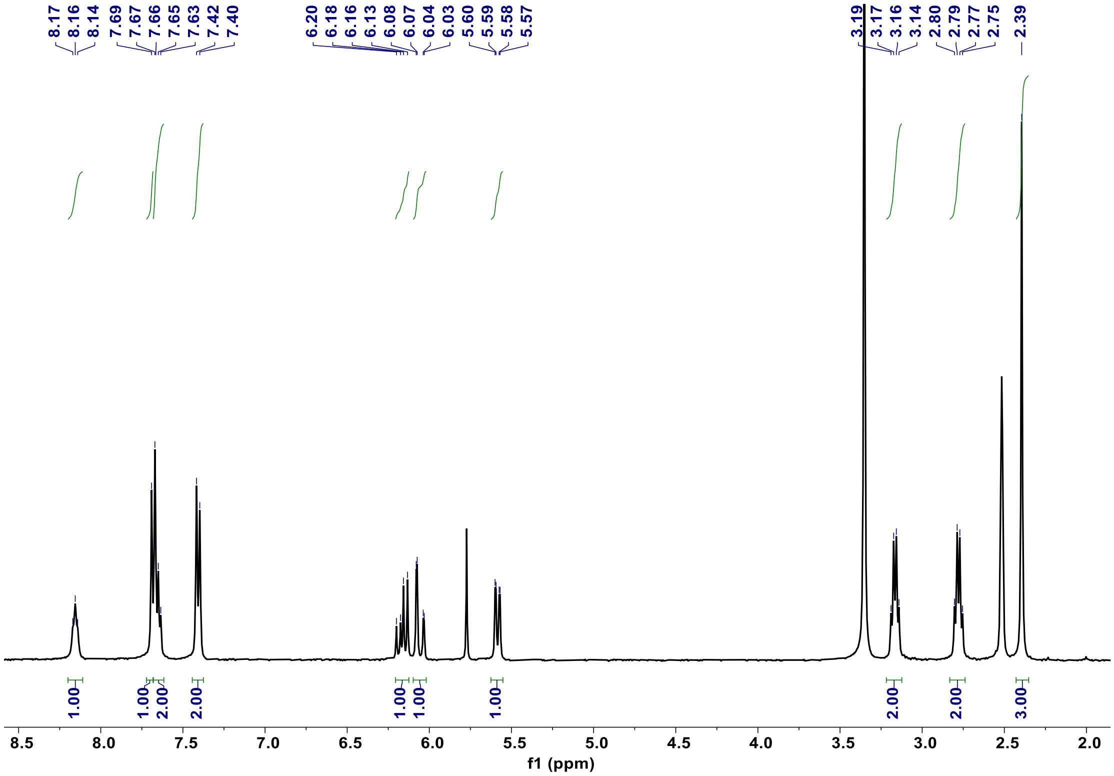

Fig. S1. ^{1}H NMR spectrum of **ETL** in DMSO-d_6, 400 MHz, 25 ˚C.

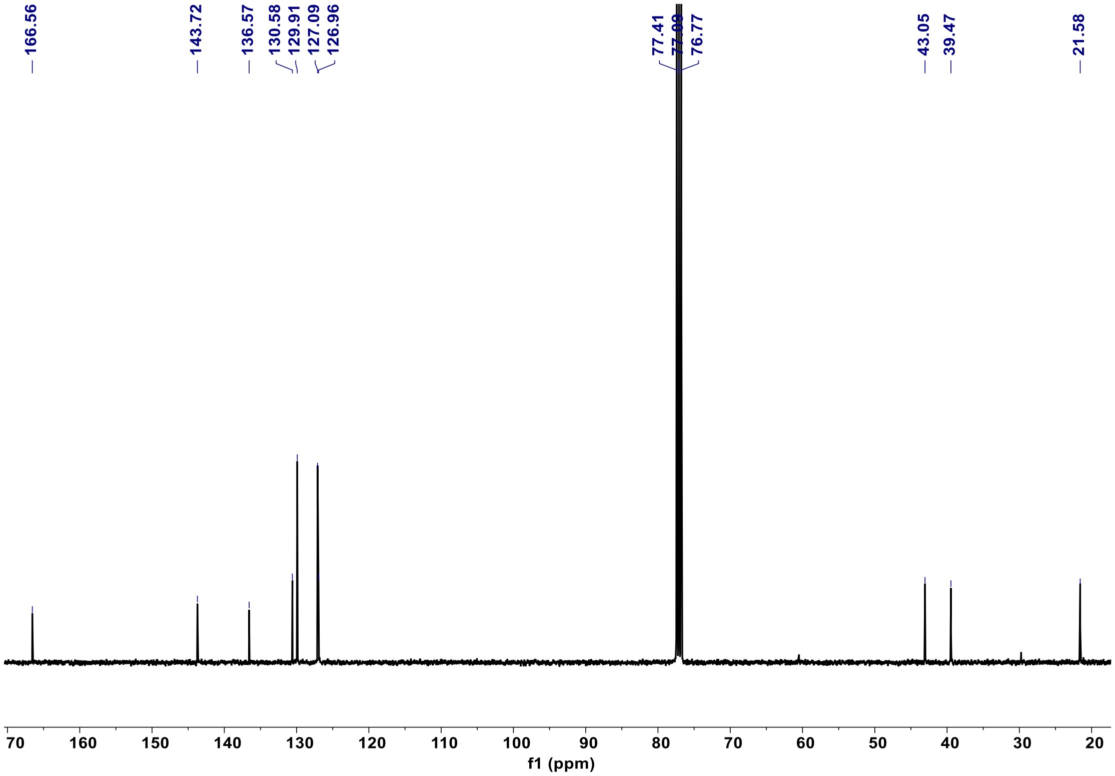

Fig. S2. ^{13}C NMR spectrum of **ETL** in $CDCl_3$.

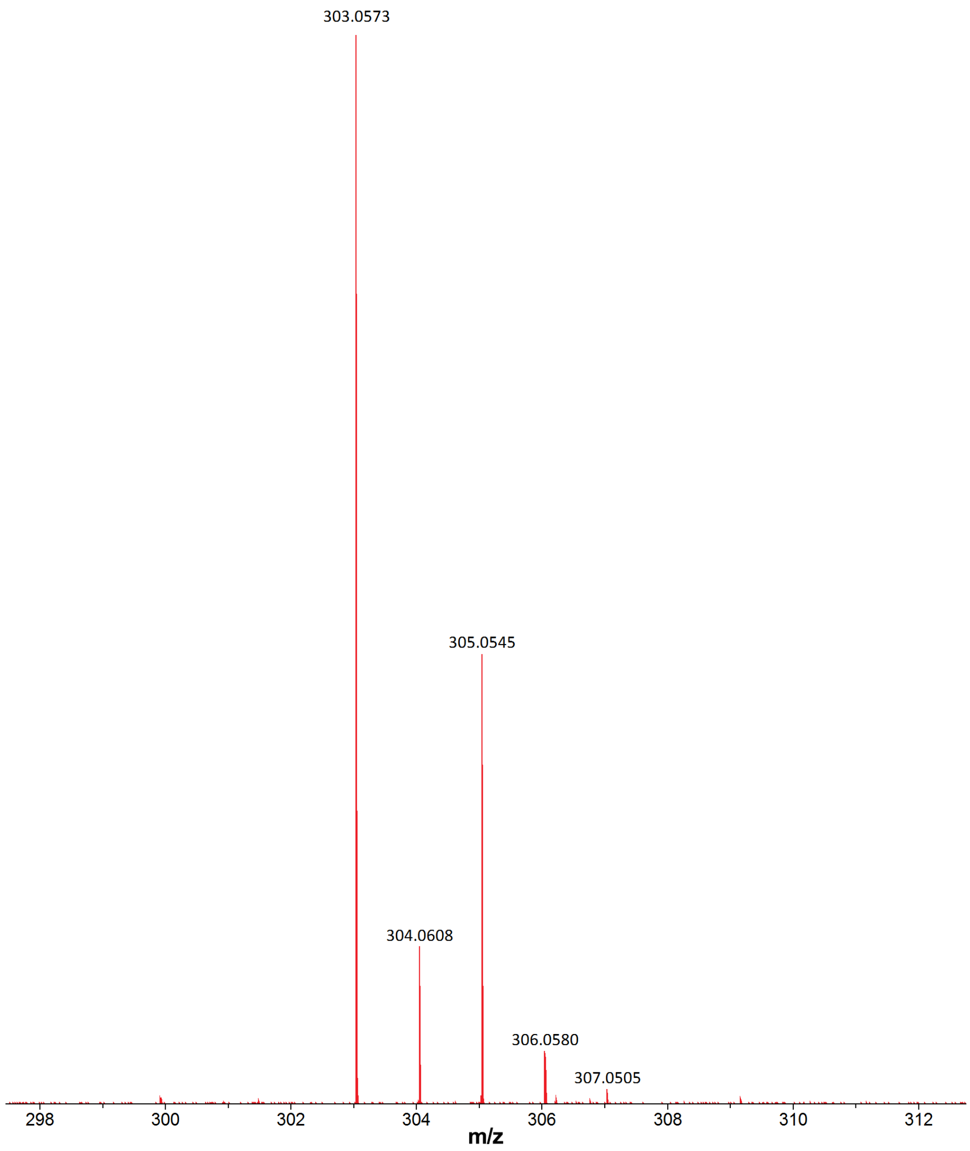

Fig. S3. Mass spectrum of ETL (ESI-MS).

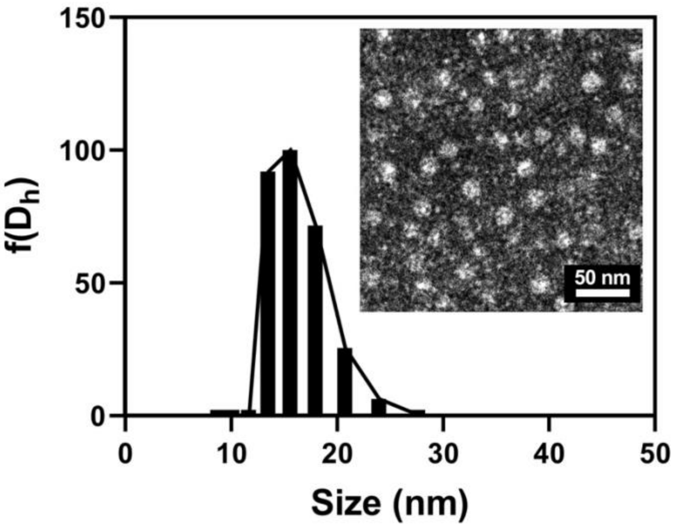

Fig. S4. Size distribution and TEM images of NanoICD/BSA. The scale bar is 50 nm.

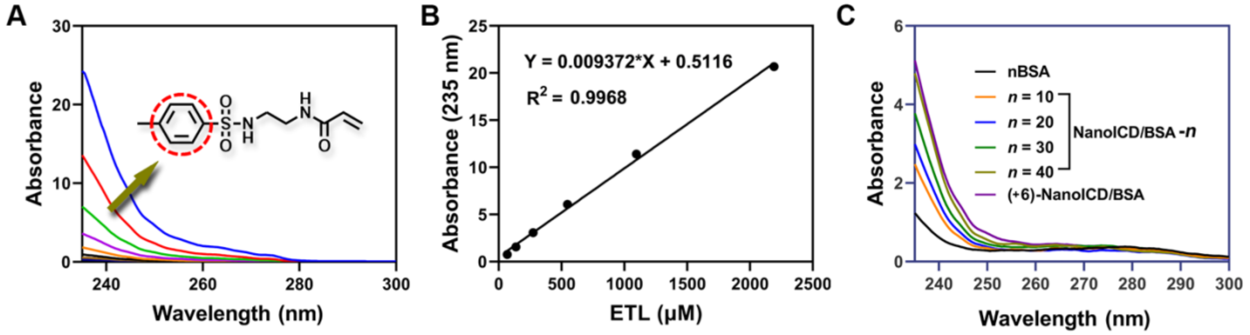

Fig. S5. **A**, UV-Vis spectra of ETL (ranging from 68.5-2192.2 μM). **B**, The standard curve of ETL. **C**, UV-Vis spectra of nBSA, NanoICD/BSA-*n* (*n* = 10, 20, 30, 40), and (+6)-NanoICD/BSA at the same concentration of BSA at 7.5 μM.

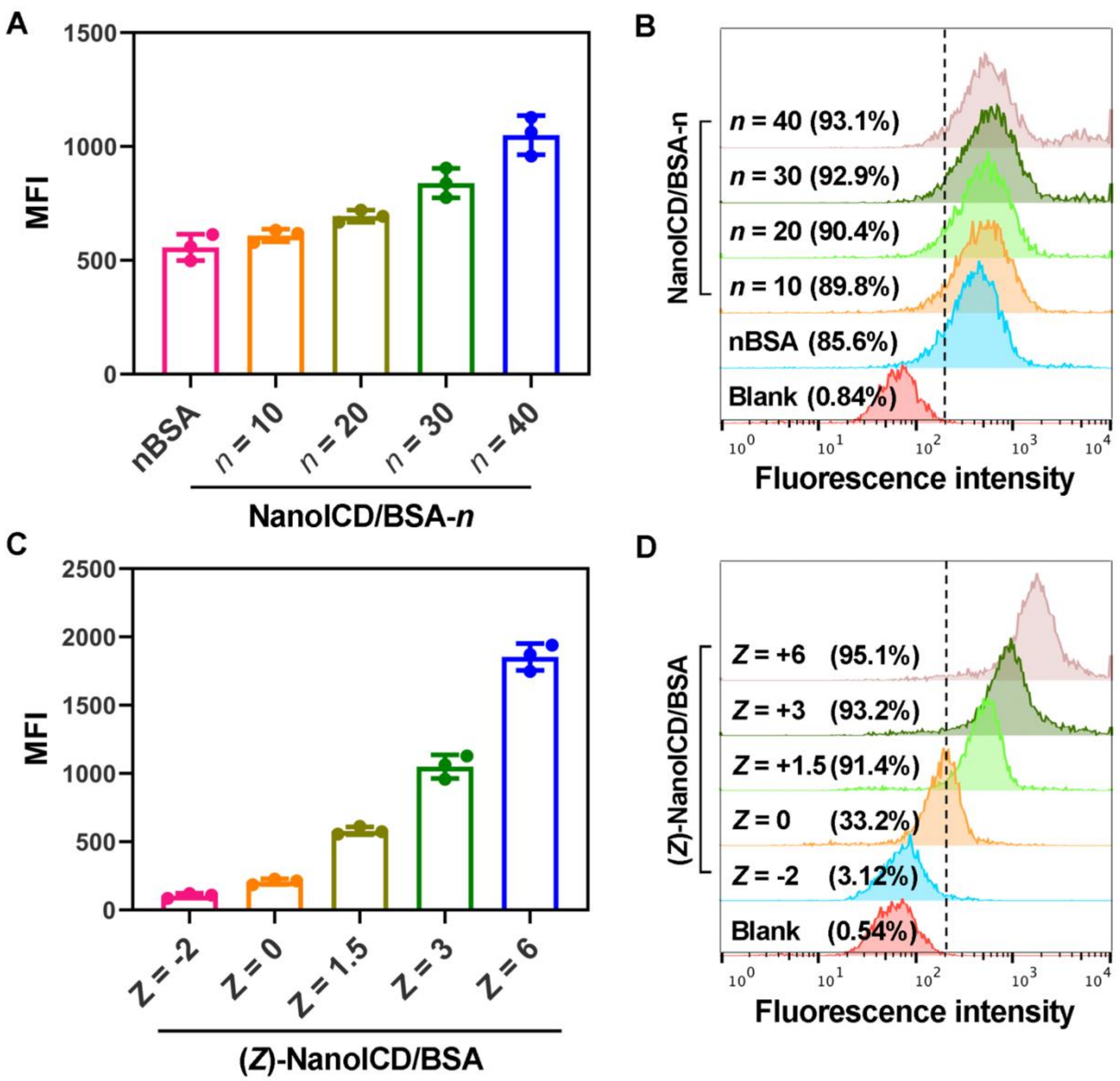

Fig. S6. Flow cytometric analysis of intracellular delivery of nBSA and NanoICD/BSA-*n* (*n* = 10, 20, 30, 40) to B16F10 after 2 h of incubation (**A**) and the corresponding representative flow cytometry plots (**B**). Flow cytometric analysis of intracellular delivery of (*Z*)-NanoICD/BSA (*Z* = -2, 0, +1.5, +3, +6) to B16F10 after 2 h of incubation (**C**) and the corresponding representative flow cytometry plots (**D**). Data are presented as mean ± s.d. from three biological replicates (*n* = 3).

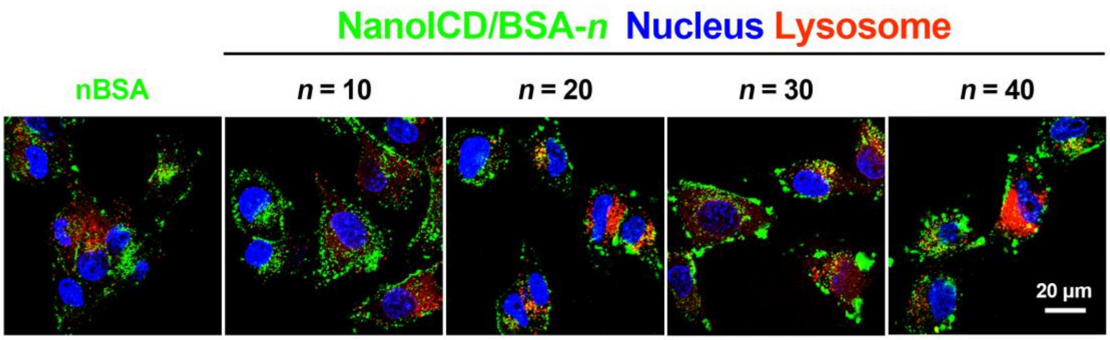

Fig. S7. Endosomal escape of nBSA and NanoICD/BSA-*n* (*n* =10, 20, 30, 40). Endosomes were stained with Lysosome-Tracker Red, and the nuclei were stained with DAPI (blue). The scare bar is 20 μm.

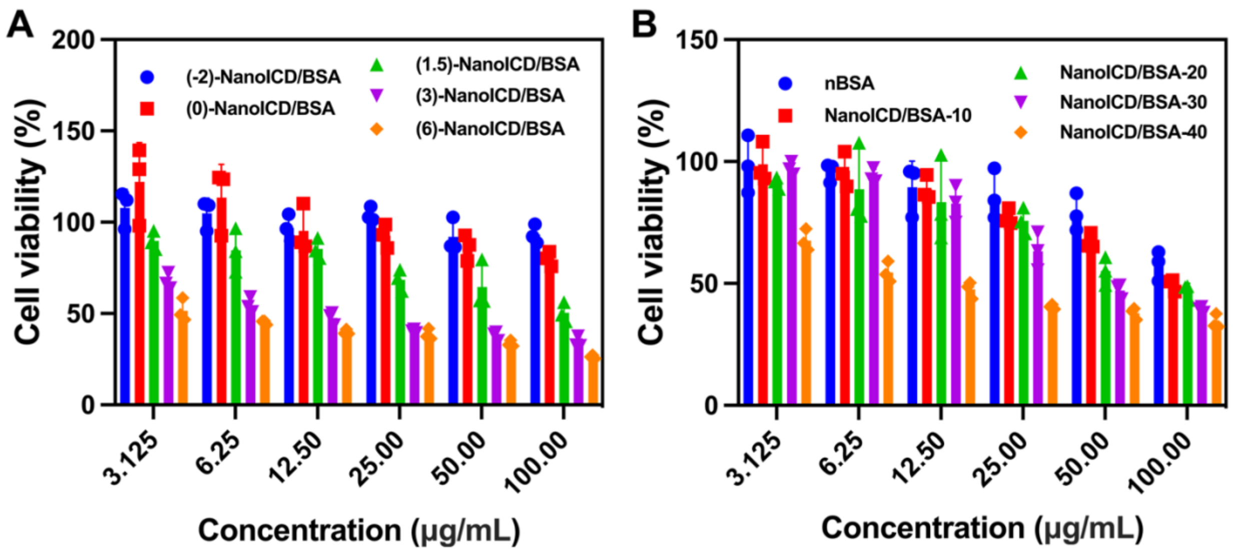

Fig. S8. The cytotoxicity of (*Z*)-NanoICD/BSA (**A**, Z = -2, 0, +1.5, +3, and +6), NanoICD-*n* (*n* = 10, 20, 30, and 40), and nBSA (**B**) to B16F10 cells. Data are presented as mean ± s.d. from three biological replicates (*n* = 3).

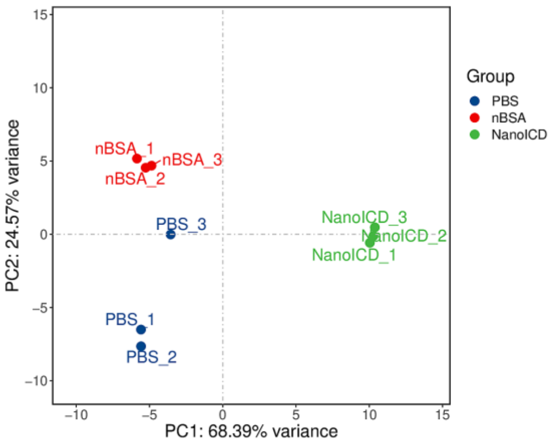

Fig. S9. Principal component analysis of B16F10 genes after treated with PBS, nBSA, and NanoICD (the NanoICD in RNA-seq analysis specially refers to NanoICD/BSA). Data are presented as mean ± s.d. from three biological replicates (*n* = 3).

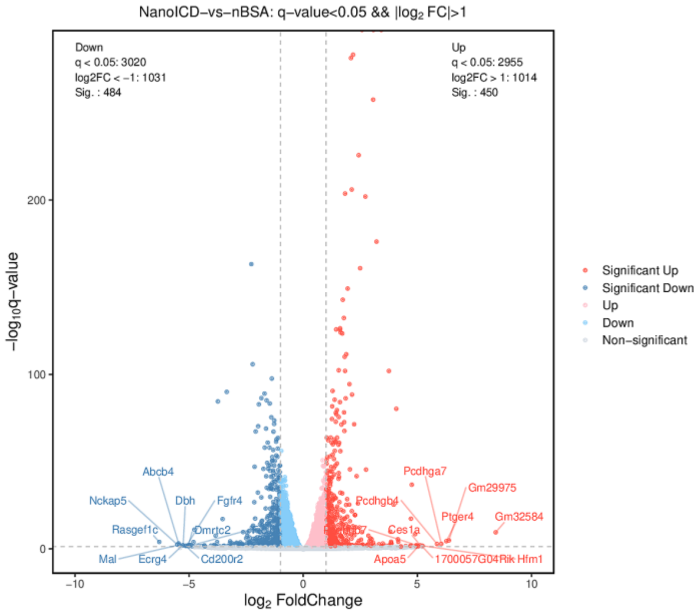

Fig. S10. Difference in gene expression [$\log_2$(fold change)] between NanoICD and nBSA-treated B16F10 cells. Genes with significantly high expression are shown in red (*P*-adj < 0.05), while genes with significantly low expression (*P*-adj < 0.05) are shown in blue. Gray denotes genes that do not exhibit significantly differences.

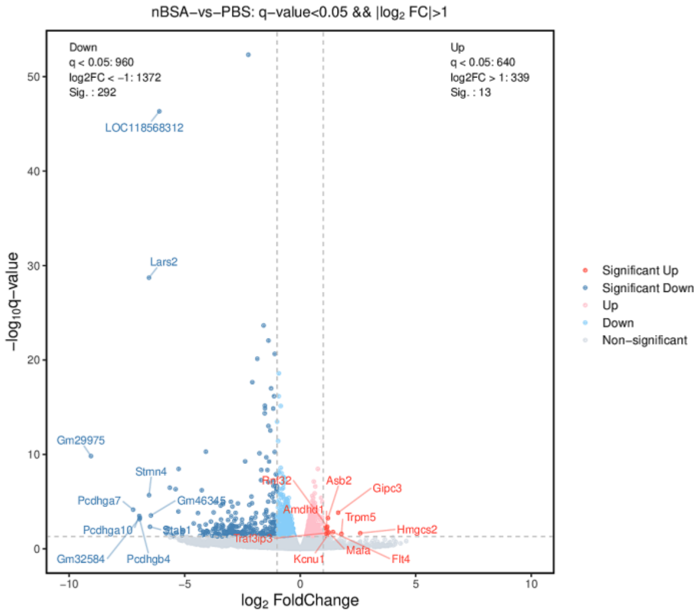

Fig. S11. Difference in gene expression [log$_2$(fold change)] between nBSA and PBS-treated B16F10 cells.

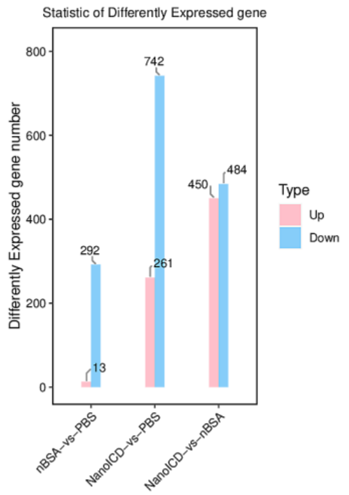

Fig. S12. Differential expressed genes (DEGs) analysis of B16F10 cell genes after treated with PBS, nBSA, and NanoICD.

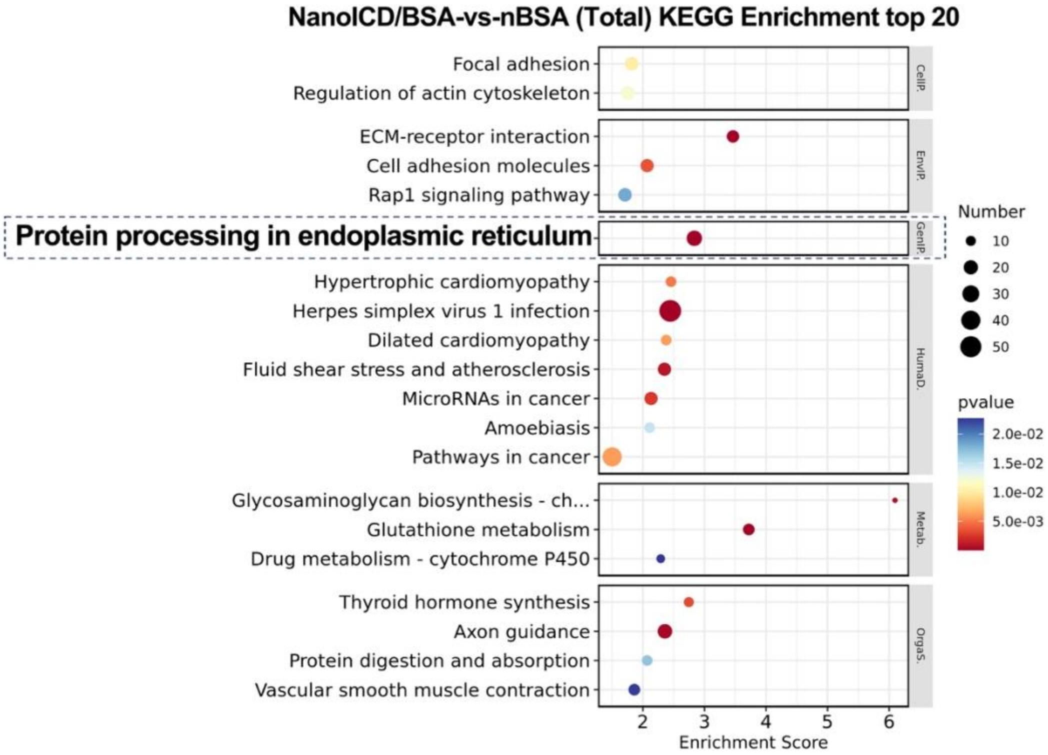

Fig. S13. Top 20 enriched pathway among KEGG analysis in NanoICD-treated B16F10 cells *vs* nBSA-treated B16F10 cells.

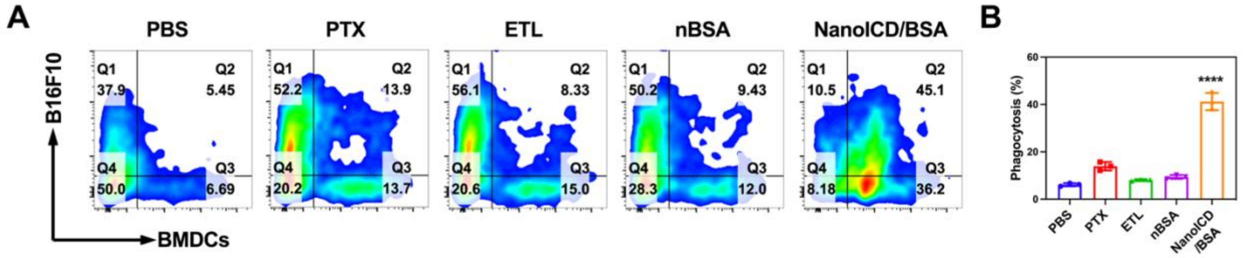

Fig. S14. Representative flow cytometry plots display the BMDC-mediated phagocytosis of B16F10 cells after being treated with PBS, PTX, ETL, nBSA, and NanoICD/BSA (**a**) and the corresponding quantitative results (**b**). Data are presented as mean ± s.d. from three biological replicates (*n* = 3).

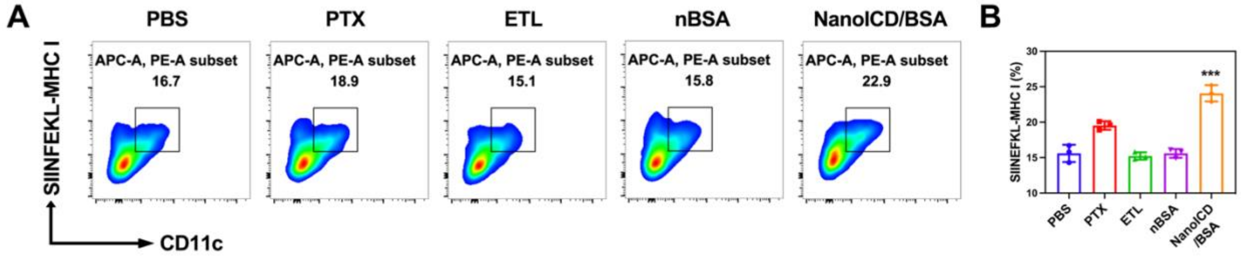

Fig. S15. Representative flow cytometry plots display the cross presentation of B16F10-OVA after being treated with PBS, PTX, ETL, nBSA, and NanoICD/BSA (**a**) and the corresponding quantitative results (**b**). Data are presented as mean ± s.d. from three biological replicates (n = 3).

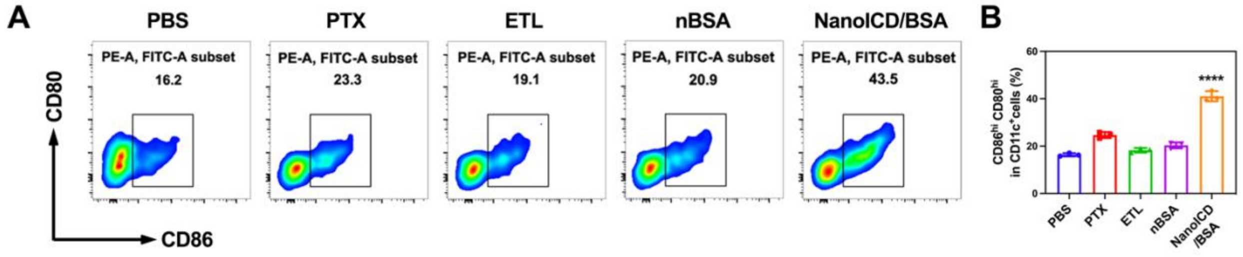

Fig. S16. Representative flow cytometry plots display the maturation of BMDC after being stimulated by PBS, PTX, ETL, nBSA, and NanoICD/BSA pre-treated B16F10 cells (**a**) and the corresponding quantitative results (**b**). Data are presented as mean ± s.d. from three biological replicates (*n* = 3).

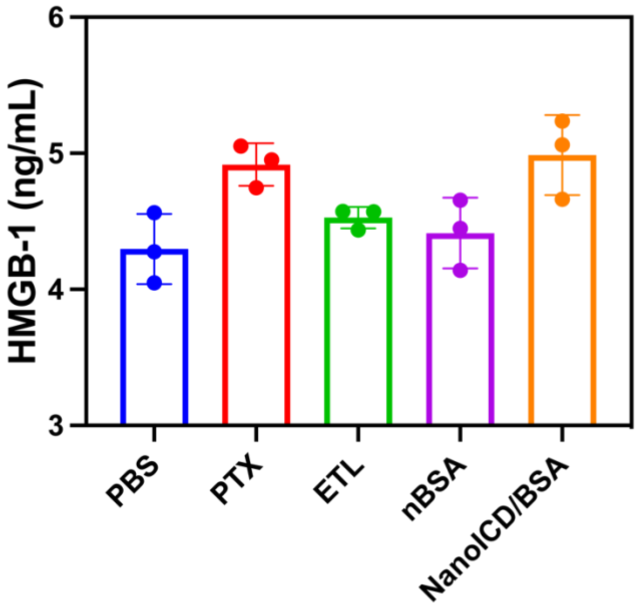

Fig. S17. Serum HMGB-1 levels in B16F10-bearing mice following treatment with PBS, PTX, ETL, nBSA, and NanoICD/BSA. Data are presented as mean ± s.d. from three biological replicates (*n* = 3).

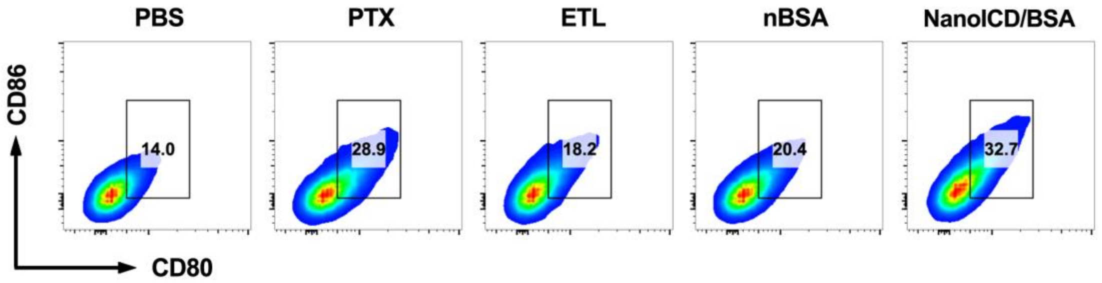

Fig. S18. Representative flow cytometry plots of mature DCs (gated on $CD80^{+}CD86^{+}$ cells) within the TDLNs from mice treated with PBS, PTX, ETL, nBSA, and NanoICD/BSA.

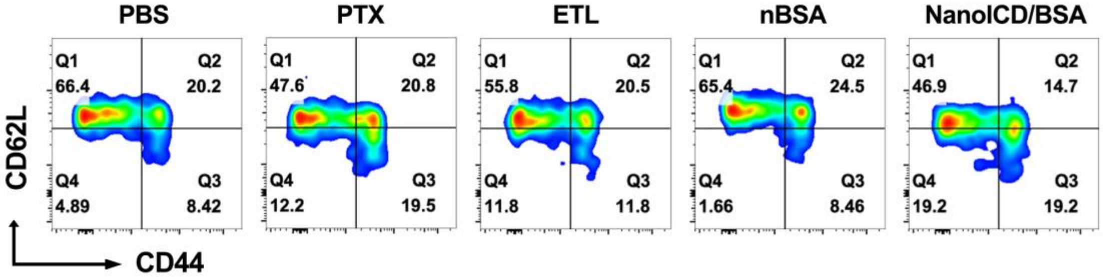

Fig. S19. Representative flow cytometry plots of effector memory cells (gating on $CD3^+CD8^+$) within the spleen from mice treated with PBS, PTX, ETL, nBSA, and NanoICD/BSA.

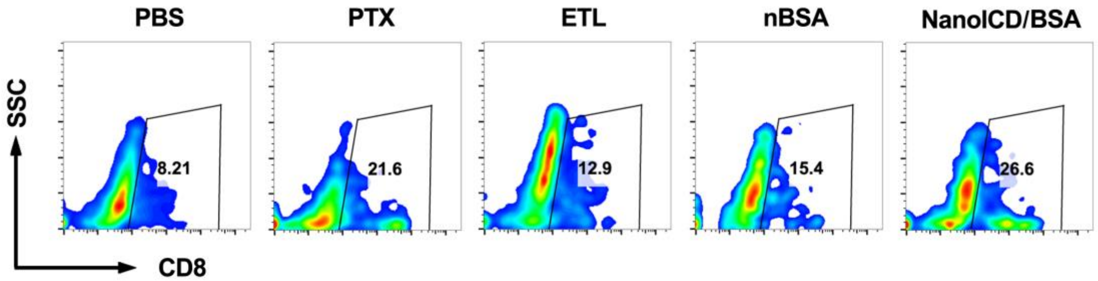

Fig. S20. Representative flow cytometry plots of $CD8^+$ T cells (gated on $CD45^+CD3^+$ cells) within the tumors from mice treated with PBS, PTX, ETL, nBSA, and NanoICD.

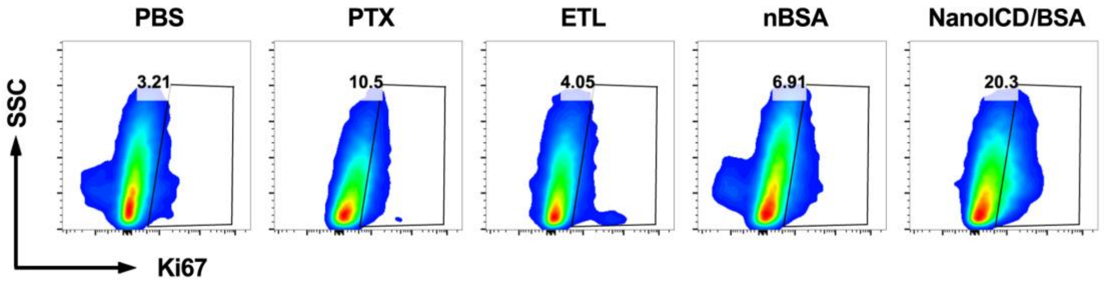

Fig. S21. Representative flow cytometry plots of Ki67$^+$ T cells (gated on CD45$^+$CD3$^+$ cells) within the tumors from mice treated with PBS, PTX, ETL, nBSA, and NanoICD.

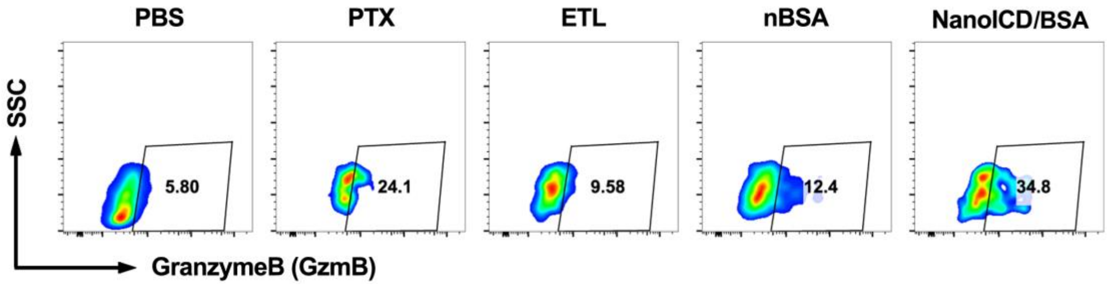

Fig. S22. Representative flow cytometry plots of GzmB$^+$CD8$^+$ T cells (gated on CD45$^+$CD3$^+$ cells) within the tumors from mice treated with PBS, PTX, ETL, nBSA, and NanoICD.

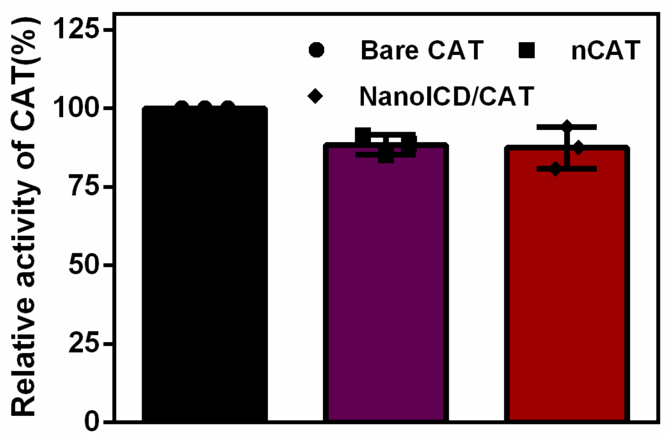

Fig. S23. Relative enzymatic activities of nCAT and NanoICD/CAT to native CAT. Data are presented as mean ± s.d. from three biological replicates (n = 3).

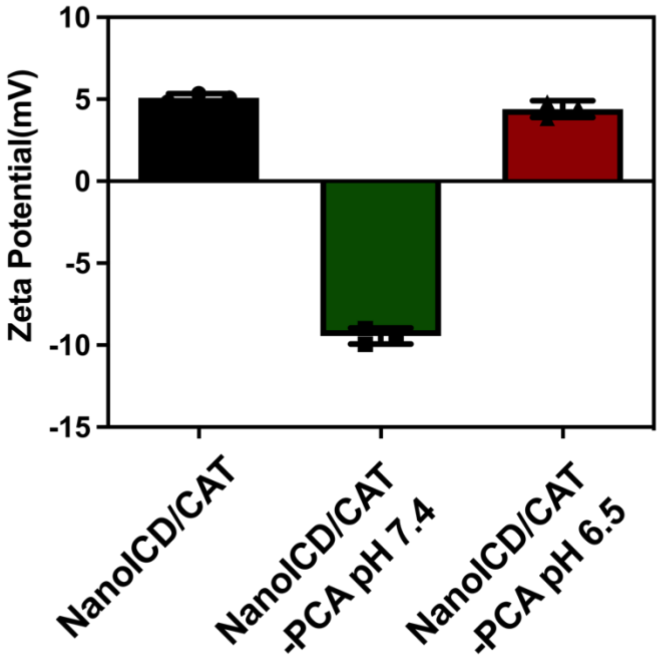

Fig. S24. Zeta potentials of NanoICD/CAT and NanoICD/CAT-PCA under different conditions. Data are presented as mean ± s.d. from three biological replicates (n = 3).

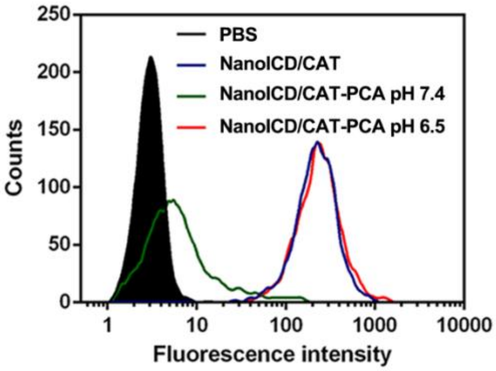

Fig. S25. Flow cytometric analysis of the 4T1 cells after incubated with NanoICD/CAT and NanoICD/CAT-PCA under different conditions.

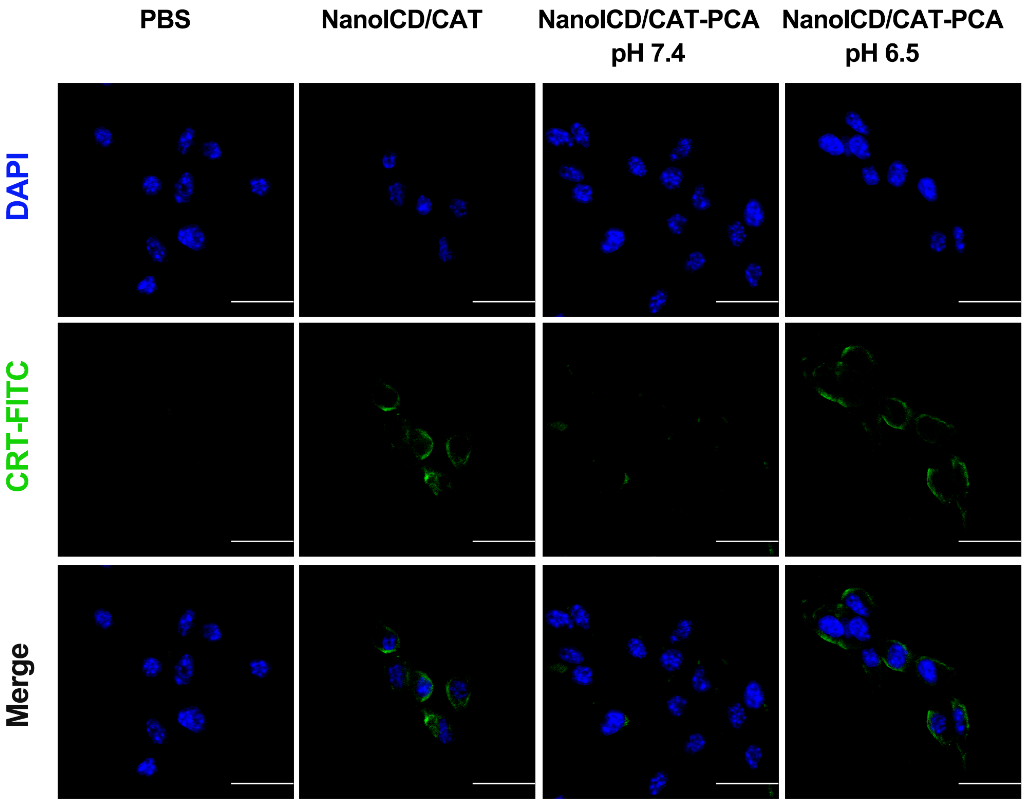

Fig. S26. CLSM images display the exposure of CRT on cell surface after treated with NanoICD/CAT and NanoICD/CAT-PCA under different conditions. The scale bars are 50 μm.

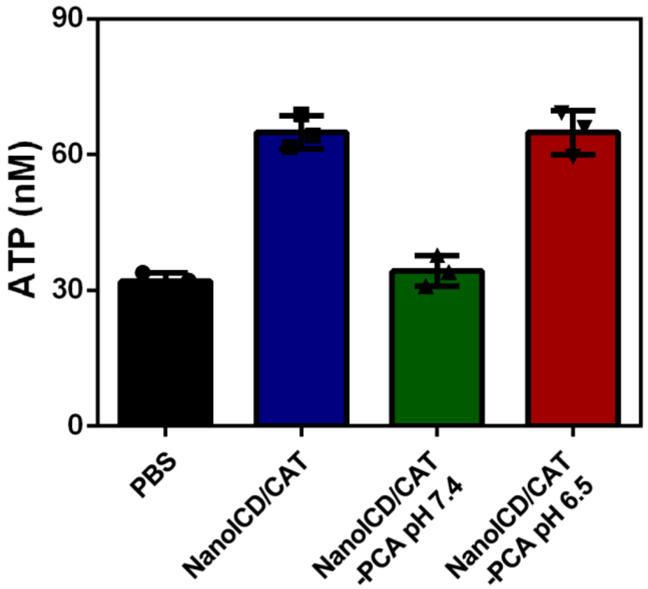

Fig. S27. The levels of ATP in cellular supernatants after different treatment. Data are presented as mean ± s.d. from three biological replicates (*n* = 3).

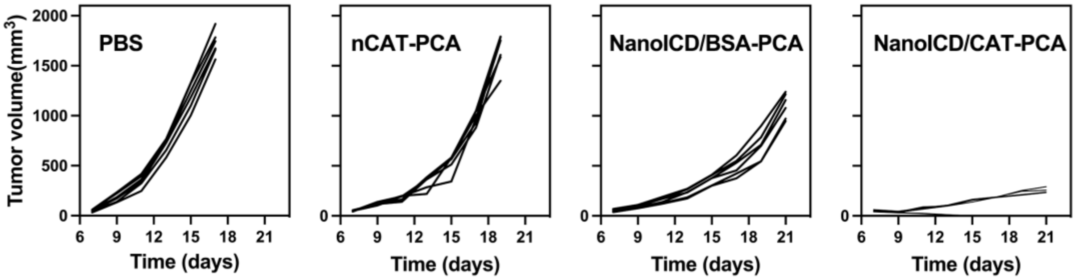

Fig. S28. Individual tumor growth kinetics in different groups. Growth curves were stopped when the first mouse of the corresponding group died.

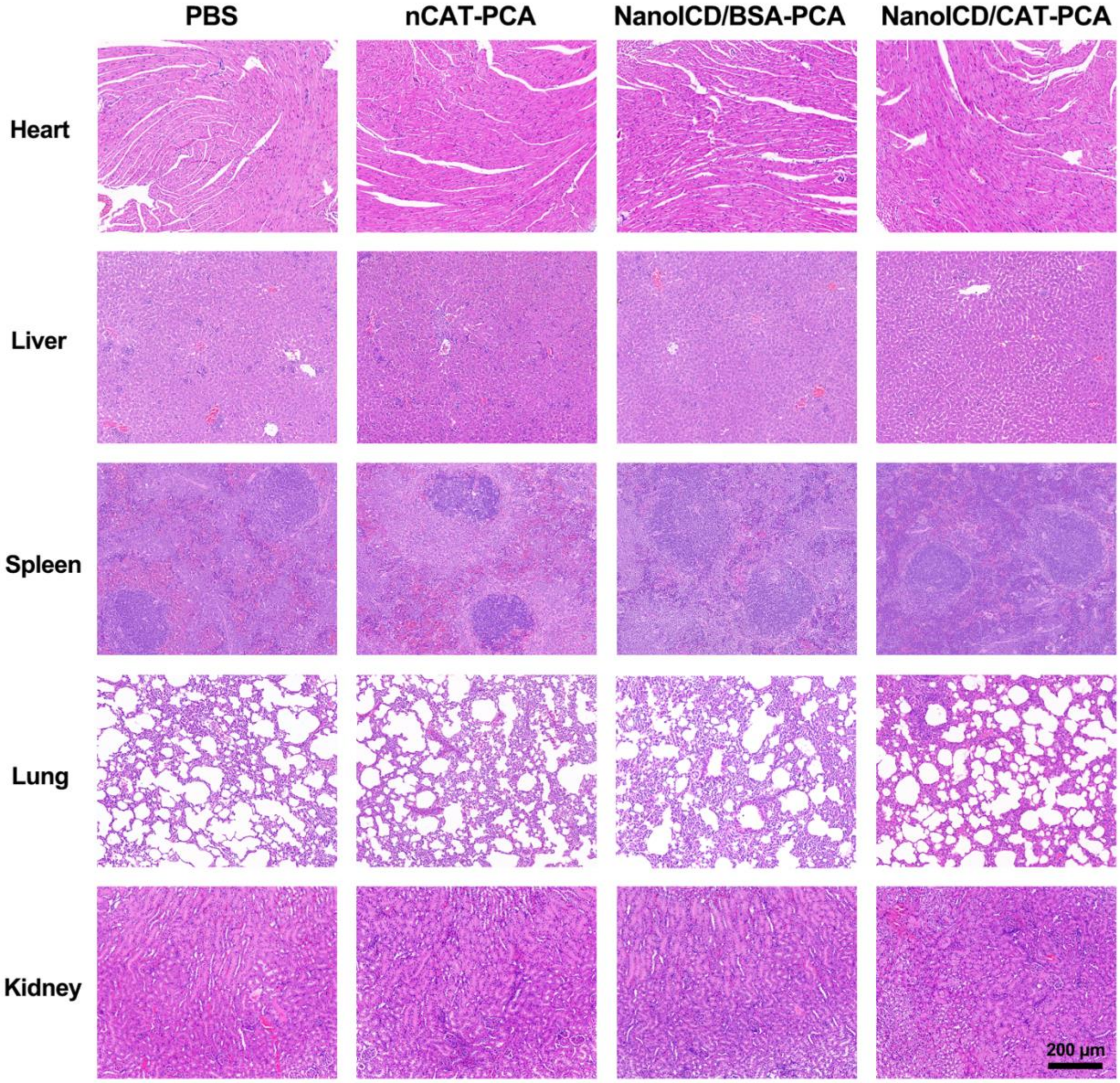

Fig. S29. H&E staining analysis of major organs from the mice in each treatment group. The scale bar is 200 µm.

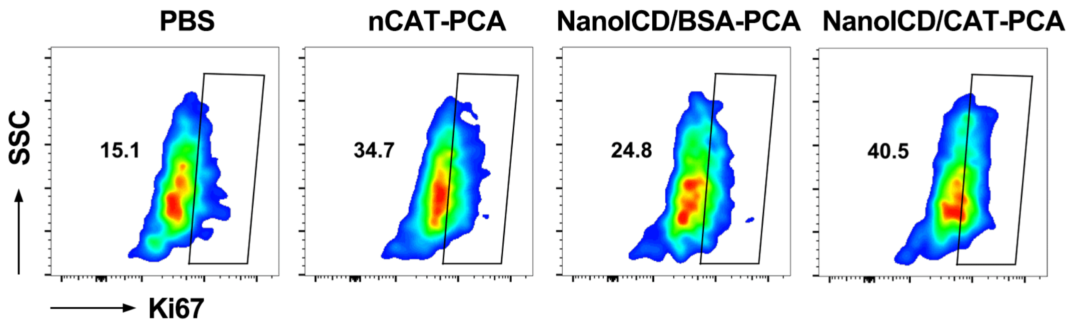

Fig. S30. Representative flow cytometry plots of Ki67$^+$ T cells (gated on CD45$^+$CD3$^+$ CD8$^+$cells) within the tumors from mice treated with PBS, nCAT-PCA, NanoICD/BSA-PCA, and NanoICD/CAT-PCA.

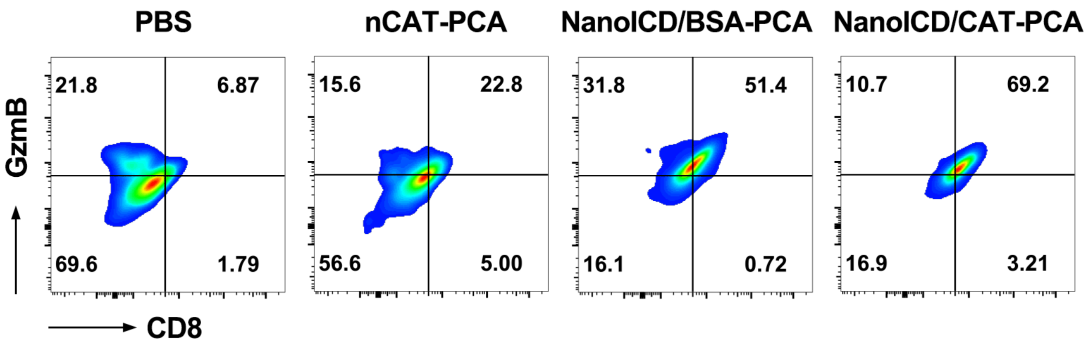

Fig. S31. Representative flow cytometry plots of $GzmB^+CD8^+$ T cells (gated on $CD45^+CD3^+$ cells) within the tumors from mice treated with PBS, nCAT-PCA, NanoICD/BSA-PCA, and NanoICD/CAT-PCA.

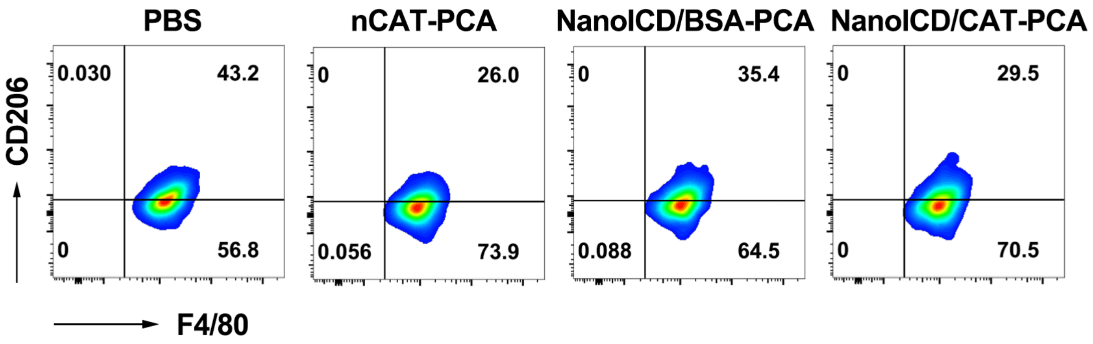

Fig. S32. Representative flow cytometry plots of M2-like TAMs (gated on $CD45^+CD11b^+$ cells) within the tumors from mice treated with PBS, nCAT-PCA, NanoICD/BSA-PCA, and NanoICD/CAT-PCA.

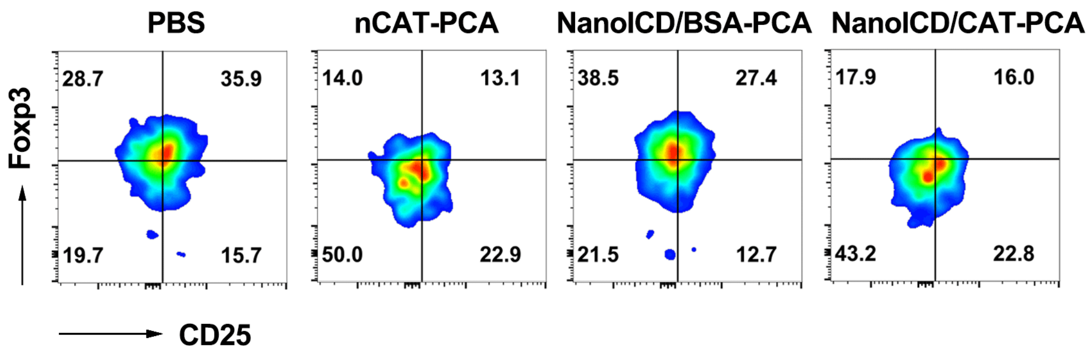

Fig. S33. Representative flow cytometry plots of Tregs (gated on $CD45^{+}CD4^{+}$ cells) within the tumors from mice treated with PBS, nCAT-PCA, NanoICD/BSA-PCA, and NanoICD/CAT-PCA.

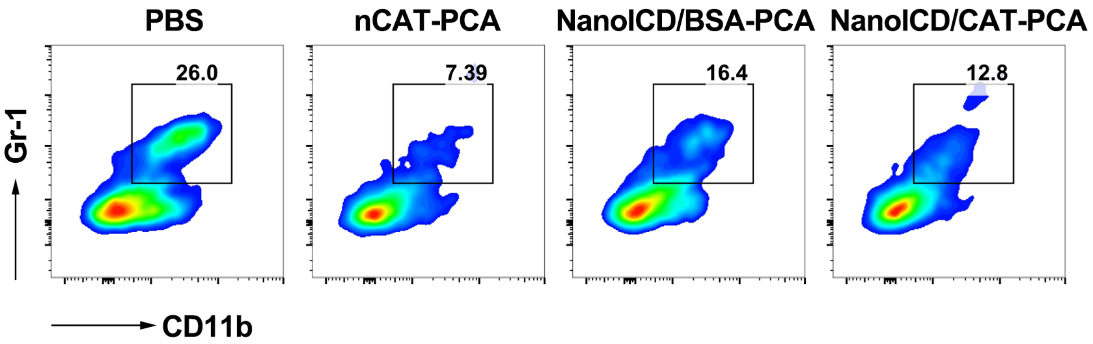

Fig. S34. Representative flow cytometry plots of MDSCs (gated on $CD45^+$ cells) within the tumors from mice treated with PBS, nCAT-PCA, NanoICD/BSA-PCA, and NanoICD/CAT-PCA.

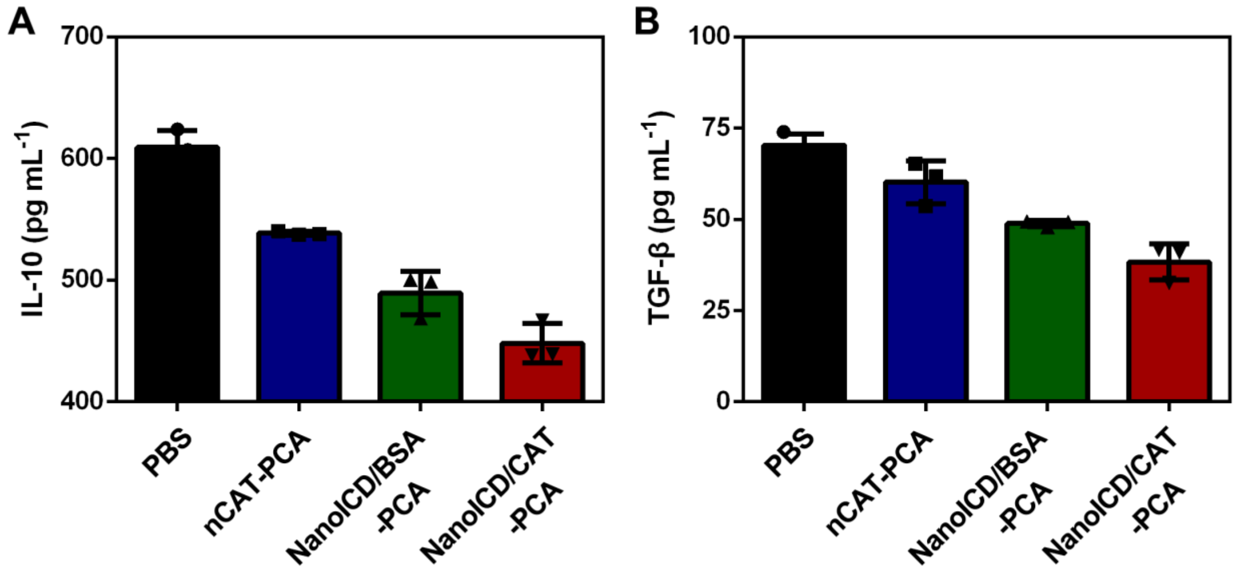

Fig. S35. IL-10 (**a**) and TGF-β (**b**) levels within the tumors from mice treated with PBS, nCAT-PCA, NanoICD/BSA-PCA, and NanoICD/CAT-PCA. Data are presented as mean ± s.d. from three biological replicates (n = 3).

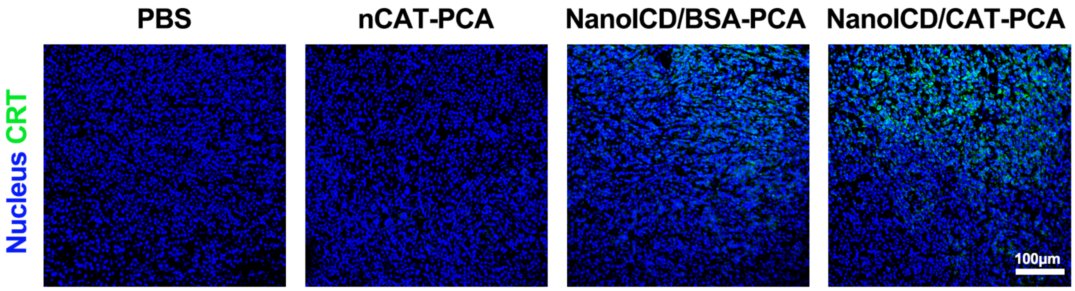

Fig. S36. Immunofluorescence staining images showing the surface translocation of CRT in tumors of mice treated with PBS, nCAT-PCA, NanoICD/BSA-PCA, and NanoICD/CAT-PCA. The scale bar is 100 μm.

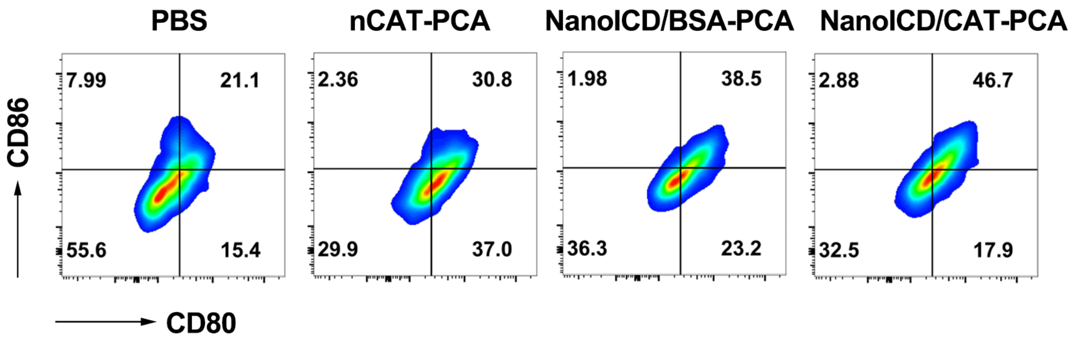

Fig. S37. Representative flow cytometry plots of mature DCs (gated on $CD80^{+}CD86^{+}$ cells) within the TDLNs from mice treated with PBS, nCAT-PCA, NanoICD/BSA-PCA, and NanoICD/CAT-PCA.

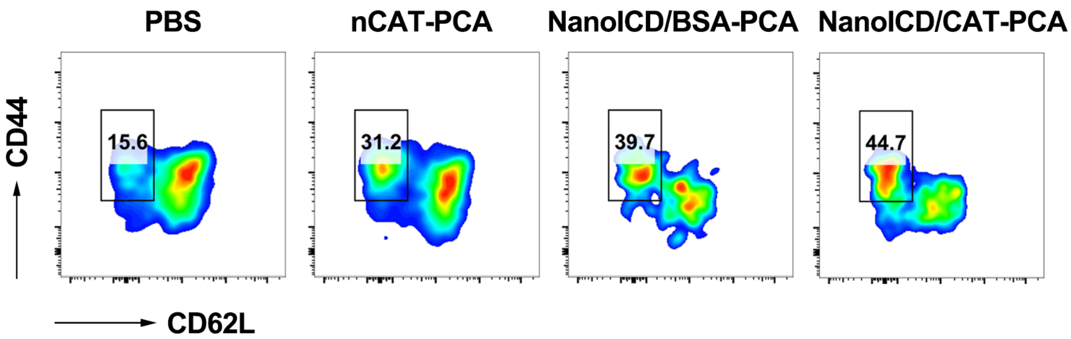

Fig. S38. Representative flow cytometry plots of effector memory cells (gating on $CD3^{+}CD8^{+}$) within the spleen from mice treated with PBS, nCAT-PCA, NanoICD/BSA-PCA, and NanoICD/CAT-PCA.

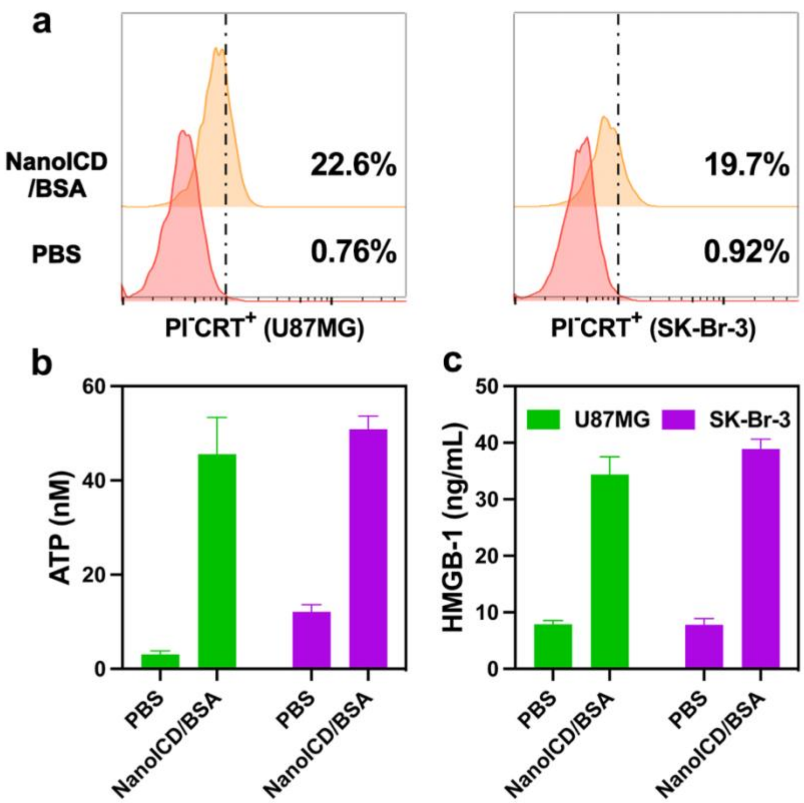

Fig. S39. The ICD-inducing activity of NanoICD/BSA in various human cell lines. **a**, Pre-apoptotic CRT exposure on cell surface (U87MG and SK-Br-3) after treatment with PBS and NanoICD/BSA. **b**, ATP levels in cellular supernatants (U87MG and SK-Br-3) after treatment with PBS and NanoICD/BSA. **c**, Extracellular concentrations of HMGB-1 after treating the cells (U87MG and SK-Br-3) with PBS and NanoICD/BSA. Data are presented as mean ± s.d. from three biological replicates (n = 3).

Table S1. The molar ratio for the preparation of different NanoICD/BSA

		BSA	AAm	APm	ETL	BIS	APS
nBSA		1	2500	500	0	300	450
NanoICD/BSA-*n*	*n* = 10	1	2500	500	50	300	450
	n = 20	1	2500	500	100	300	450
	n = 30	1	2500	500	150	300	450
	n = 40	1	2500	500	200	300	450
(6)-NanoICD/BSA		1	2500	750	200	300	450

Table S2. The molar ratio for the preparation of NanoICD/CAT

	CAT	AAm	APm	ETL	BIS	APS
nCAT	1	3500	550	0	420	350
NanoICD/CAT	1	3500	550	200	420	350

Table S3. Characterization of different NanoICD and the pro-apoptotic CRT exposure on cell surface (PI^-CRT^+ cells) after treatment

Sample		Amount of ETL	Size (nm)	Zeta potential (mV)	PDI	PI^- CRT^+ (%)
nBSA		-	15.2 ± 1.79	3.0 ± 0.30	0.236	1.55
NanoICD/BSA-*n*	*n* = 10	10.5 ± 0.73	19.5 ± 1.77	2.82 ± 0.39	0.214	1.75
	n = 20	20.6 ± 2.12	15.7 ± 1.83	3.43 ± 0.52	0.173	2.91
	n = 30	31.2 ± 2.32	17.2 ± 1.24	3.21 ± 0.35	0.223	24.0
	n = 40	41.2 ± 2.39	16.9 ± 0.73	3.05 ± 0.15	0.117	38.3
(*Z*)-NanoICD/BSA	*Z* = -2	41.2 ± 2.39	16.9 ± 0.73	-1.93 ± 0.30	0.117	1.41
	Z = 0	41.2 ± 2.39	16.9 ± 0.73	0.03 ± 0.44	0.117	2.33
	Z = 1.5	41.2 ± 2.39	16.9 ± 0.73	1.56 ± 0.13	0.117	14.2
	Z = 6	42.0 ± 1.45	17.5 ± 1.78	6.32 ± 0.56	0.213	5.55
nCAT		-	19.6 ± 1.23	3.78 ± 0.30	0.128	1.91
NanoICD/CAT		40.2 ± 1.74	22.5 ± 1.56	5.40 ± 0.55	0.230	25.80

Table S4. Abbreviations

Abbreviations	Full names	ETL amount	Zeta potential
nBSA	Nanoparticles incorporating APm onto BSA surface	0	+3 mV
nCAT	Nanoparticles incorporating APm onto CAT surface	0	+3 mV
NanoICD/BSA	Nanoparticles incorporating APm and ETL onto BSA surface	40	+3 mV
NanoICD/CAT	Nanoparticles incorporating APm and ETL onto CAT surface	40	+3 mV
NanoICD/BSA-10	NanoICD/BSA with 10 ETL integrated onto its surface	10	+3 mV
NanoICD/BSA-20	NanoICD/BSA with 20 ETL integrated onto its surface	20	+3 mV
NanoICD/BSA-30	NanoICD/BSA with 30 ETL integrated onto its surface	30	+3 mV
NanoICD/BSA-40	NanoICD/BSA with 40 ETL integrated onto its surface	40	+3 mV
(-2)-NanoICD/BSA	NanoICD/BSA with a zeta potential of -2 mV	40	-2 mV
(0)-NanoICD/BSA	NanoICD/BSA with a zeta potential of 0 mV	40	0 mV
(+3)-NanoICD/BSA	NanoICD/BSA with a zeta potential of +3 mV	40	+3 mV
(+6)-NanoICD/BSA	NanoICD/BSA with a zeta potential of +6 mV	40	+6 mV
nCAT-PCA	nCAT that coated with PCA	0	-10 mV
NanoICD/CAT-PCA	NanoICD/CAT that coated with PCA	40	-10 mV
